# Broad-Spectrum Antiviral Activity of Pyridobenzothiazolone Analogues Against Respiratory Viruses

**DOI:** 10.3390/v17070890

**Published:** 2025-06-24

**Authors:** Elisa Feyles, Tommaso Felicetti, Irene Arduino, Massimo Rittà, Andrea Civra, Luisa Muratori, Stefania Raimondo, David Lembo, Giuseppe Manfroni, Manuela Donalisio

**Affiliations:** 1 Laboratory of Molecular Virology and Antiviral Research, Department of Clinical and Biological Sciences, University of Turin, Regione Gonzole 10, 10043 Orbassano, Italy; elisa.feyles@unito.it (E.F.); irene.arduino@unito.it (I.A.); massimo.ritta@unito.it (M.R.); andrea.civra@unito.it (A.C.); david.lembo@unito.it (D.L.); 2 Department of Pharmaceutical Sciences, University of Perugia, Via del Liceo, 1, 06123 Perugia, Italy; tommaso.felicetti@unipg.it (T.F.); giuseppe.manfroni@unipg.it (G.M.); 3 Department of Clinical and Biological Sciences, Neuroscience Institute Cavalieri Ottolenghi (NICO), University of Turin, Regione Gonzole 10, 10043 Orbassano, Italy; luisa.muratori@unito.it (L.M.); stefania.raimondo@unito.it (S.R.)

**Keywords:** antiviral agents, broad-spectrum activity, Pyridobenzothiazolones, respiratory tract infections

## Abstract

Cell-based phenotypic screening of a privileged in-house library composed of pyridobenzothiazolone (PBTZ) analogues was conducted against representative viruses responsible for common respiratory tract infections in humans, i.e., respiratory syncytial virus (RSV), human coronavirus type OC43 (HCoV-OC43), and influenza virus type A (IFV-A). We identified a compound with broad-spectrum inhibitory activity against multiple strains of RSV, HCoV, and IFV, with EC_50_ values in the low micromolar range and cell-independent activity. Its antiviral activity and cytocompatibility were confirmed in a fully differentiated 3D model of the bronchial epithelium mimicking the in vivo setting. The hit compound enters cells and localizes homogeneously in the cytosol, inhibiting replicative phases in a virus-specific manner. Overall, the selected PBTZ represents a good starting point for further preclinical development as a broad-spectrum antiviral agent that could address the continuous threat of new emerging pathogens and the rising issue of antiviral resistance.

## 1. Introduction

Every year, respiratory infectious diseases cause high morbidity rates in humans, severely impacting healthcare systems, societies, and economies worldwide. In particular, respiratory viruses—especially coronavirus, respiratory syncytial virus, and influenza—are responsible for millions of hospitalizations and deaths among children in low-income countries, immunocompromised individuals, pregnant women, and the elderly. These viruses may cause both upper and lower tract symptoms with variable severity. Notably, mild symptoms in healthy individuals can exacerbate into severe respiratory tract conditions in at-risk populations, leading to serious outcomes and favoring the onset of chronic diseases [1,2]. Respiratory infections are usually treated with supportive care, as only a few antivirals are licensed for therapy, and these are usually virus-specific (e.g., oseltamivir or baloxavir for influenza virus, Paxlovid for coronavirus) and frequently limited by safety and bioavailability issues [3,4,5]. Moreover, the high viral genome mutation rate, rapid airborne transmission and replication, and selective pressure exerted by antivirals all contribute to the emergence of drug resistance, in turn resulting in the inefficacy of approved antiviral drugs. In this context, there is an urgent medical need to develop novel antiviral molecules that target multiple viruses belonging to various families (direct-acting antiviral drugs) or cellular pathways critical for the infection and replication of multiple viruses (host-targeted antiviral drugs) [6]. These so-called broad-spectrum antiviral agents (BSAAs) are expected to address the rising frequency of viral epidemic/pandemic outbreaks and are necessary to rapidly and proficiently treat patients with infections.

Herein, we undertook an evaluation of the broad-spectrum antiviral potential of a library of 29 pyridobenzothiazolone (PBTZ) analogues, previously investigated for their anti-flavivirus potential [7], against three common respiratory viruses: human coronavirus type OC43 (HCoV-OC43), influenza virus type A H1N1 (IFV-A H1N1), and respiratory syncytial virus type A (RSV-A2). The three enveloped viruses were selected as representatives of common human respiratory viruses, with different genome structures and replicative strategies (ssRNA(+): HCoV, ssRNA(-): IFV and RSV).

## 2. Materials and Methods

### 2.1. Cell Lines and Human-Derived Epithelia

Human lung fibroblast cells (MRC-5; ATCC^®^ CCL-171™, Manassas, VA, USA), human epithelial cells (Hep-2; ATCC^®^ CCL-23™, Manassas, VA, USA), Madin–Darby canine kidney cells (MDCK; ATCC^®^ CCL-34™, Manassas, VA, USA), and human lung carcinoma epithelial cells (A549; ATCC^®^ CCL-185™, Manassas, VA, USA) were cultured using Dulbecco’s modified Eagle’s medium (DMEM; Sigma-Aldrich, Merck Life Science S.r.l., Milan, Italy) supplemented with 10% (*v*/*v*) heat-inactivated fetal bovine serum (FBS; Thermo Fisher Scientific, Waltham, MA, USA) and with 1% (*v*/*v*) antibiotic/antimycotic solution (Sigma-Aldrich, Merck Life Science S.r.l., Milan, Italy). The cells were grown at 37 °C in a humidified 5% CO_2_ incubator. Three-dimensional fully differentiated epithelia derived from a mixture of human bronchial cells isolated from a single donor (MucilAir™, EP01MD) were purchased from Epithelix Sàrl (Geneva, Switzerland). The tissue inserts were cultured at the air–liquid interface (ALI) using MucilAir™ culture medium (EP05MM), in 24-well plates with 6.5 mm Transwell^®^ inserts (cat #3470, Corning Incorporated, Corning, NY, USA), at 37 °C in a humidified 5% CO_2_ atmosphere, according to the supplier’s instructions.

### 2.2. Viruses

Human betacoronavirus type OC43 (HCoV-OC43; ATCC^®^ VR-1558™, Manassas, VA, USA) and human alphacoronavirus type 229E (HCoV-229E, 0310051v; ECACC, Salisbury, Wiltshire, England) were produced in MRC-5 cells. Influenza viruses, type A, including H1N1 (IFV-A H1N1; strain A/California/07/2009 (H1N1) pdm09; ATCC^®^ VR-1894™) and H3N2 strains (IFV-A H3N2; strain A/ChristChurch/28/03; Italian National Institute of Health, Rome, Italy), and type B Victoria lineage (IFV-B; strain B/Florida/78/2015; ATCC^®^ VR-1931™) were propagated in MDCK cells in DMEM containing 1 μg/mL of IX-type porcine pancreatic trypsin (Sigma-Aldrich, Merck Life Science S.r.l.). Respiratory syncytial viruses—strain A2 (RSV-A2; ATCC^®^ VR-1540™) and strain B WV/14617/85 (RSV-B; ATCC^®^ VR-1400™)—were produced in Hep-2 cells. When the cytopathic effect (CPE) occurred, cell culture supernatants were harvested, clarified, aliquoted, and stored at −80 °C. Titers of the viral stocks were determined via a focus assay (for HCoV and IFV) or plaque assay (for RSV). Briefly, the cells were infected with serial dilutions of the virus suspension, and at 16 (for HCoV and IFV) or 72 (for RSV) hours post-infection (h.p.i.), the cells were subjected to indirect immunocytochemistry. Briefly, the cells were fixed with cold acetone/methanol (50:50), permeabilized with PBS 1X-Triton X-100 0.1%, incubated with primary antibodies directed against specific viral antigens and secondary antibodies conjugated to horseradish peroxidase (HRP), and stained by adding 3,3′ diaminobenzidine tetrahydrochloride (DAB Substrate; 11,718,096,001, Merck Life Science S.r.l.). Brown-stained infective events were counted using an optical microscope, and titers were expressed as focus-/plaque-forming unit per mL (FFU/mL and PFU/mL). Virus stocks and all subsequent cell-based experiments were performed with DMEM supplemented with 2% (*v*/*v*) FBS in a humidified 5% CO_2_ atmosphere at 34 °C (for HCoVs, IFV-B) or at 37 °C (for RSV and IFV-A).

### 2.3. Chemistry

All details on the general chemical and experimental procedures for the synthesis of the target compounds are reported in the Supplementary Materials, as previously reported [7,8,9,10,11].

### 2.4. Virus Inhibition Assay

The antiviral activity of the compounds was assessed via focus (for HCoV and IFV) or plaque (for RSV) reduction assays, as previously described [12,13]. Briefly, sub-confluent cells seeded in 96-well plates were pretreated with serial dilutions of a compound for 2 h; then, a fixed viral inoculum (multiplicity of infection, FFU/cell or PFU/cell, MOI: 0.01 for HCoV, IFV; 0.001 for RSV) and serial dilutions of the compound were added. HCoV- and IFV-infected cells were incubated for 20 h at 34 °C (HCoV, IFV-B) or 37 °C (IFV-A). RSV-infected cells were incubated with a virus/compound mixture for 2 h and overlaid with medium containing 1.2% methylcellulose and serial dilutions of the compound for 72 h at 37 °C. After incubation, the cells were fixed and subjected to immunocytochemistry, as reported above. Infected foci/plaques were quantified, and viral infectivity was reported as the mean percentage of the untreated control ± standard error of the mean (SEM).

### 2.5. Cell Viability and Cytotoxicity Assays

Cell viability was measured with the MTS [3-(4,5-dimethylthiazol-2-yl)-5-(3-carboxymethoxyphenyl)-2-(4-sulfophenyl)-2H-tetrazolium] assay, using the Cell Titer 96 Proliferation Assay Kit on 96-well plates as previously reported [14]. Cells were seeded in 96-well plates and treated with serial dilutions of the compounds on the following day, under the same experimental conditions as the virus inhibition assays. Absorbances were measured at 490 nm using a Microplate Reader (Model680, BIORAD, Hercules, CA, USA). The effect of the compounds on the cell viability of all used cell lines is expressed as a percentage, determined by comparing the absorbances of treated cells with those of cells incubated with an equal amount of DMSO as a control. Cytotoxicity was assessed via the lactate dehydrogenase (LDH) assay using the CytoTox 96 Non-Radioactive Cytotoxicity Assay kit (Promega, Madison, WI, USA), as previously described [13].

### 2.6. Virus Inactivation Assay

The virucidal activity of compound **1** was evaluated against HCoV-OC43 and RSV-A2. An EC_90_ dose of the compound and 200,000 FFUs/PFUs of the virus were mixed together in a total volume of 100 µL and incubated for 2 h. Then, the mixture was serially diluted to the non-inhibitory concentration of compound **1**, and the residual infectivity was determined with the focus/plaque reduction assay, as described above.

### 2.7. Time-of-Addition Assay

To investigate the mechanism of action of compound **1**, cells were treated with serial dilutions of the compound for 2 h before viral infection (pre-treatment), for 2 h during viral infection (co-treatment), or after virus inoculum removal (post-treatment) and then treated as for the virus inhibition assay. The number of foci/plaques was determined after 16 h (HCoV-OC43) or 72 h (RSV-A2), and viral infectivity was reported as the mean percentage of the untreated control ± SEM.

### 2.8. Cellular Internalization via Confocal Laser Microscopy

The determination of the λmax of the fluorescence emission of compound **1** (autofluorescence) is reported in the SI. Hep-2 and MRC-5 cells pre-seeded in 24-well plates on coverslips were treated with compound **1** at the respective EC_90_ doses for RSV-A2 (Hep-2) and HCoV-OC43 (MRC-5) or with equal amounts of DMSO for the control. After 1 h, 3 h, or 24 h of treatment, the cells were washed with sterile PBS 1X five times. Images of live cell sections were acquired with a confocal laser microscope (LSM800, Carl Zeiss, Jena, Germany) and analyzed using Zen Software, 3.7 version (Carl Zeiss).

### 2.9. Immunofluorescence Experiments

Pre-seeded sub-confluent Hep-2 and MRC-5 cells on coverslips were infected with RSV and HCoV-OC43 (MOI: 0.1), respectively, and concurrently treated with the respective EC_90_s of compound **1**. After 24 h of incubation, the cells were washed twice with PBS and fixed in 4% paraformaldehyde for 15 min at room temperature. Immunostaining was performed as previously described [13], with the anti-dsRNA primary antibody (RNT-SCI-10010200, Jena Bioscience, Jena, Germany), the anti-RSV primary antibody (ab43812, Abcam, Cambridge, UK), or the anti-OC43 nucleoprotein primary antibody (MAB9013, Sigma-Aldrich, Merck Life Science S.r.l.), followed by incubation with the secondary antibody (goat anti-mouse Alexa Fluor™ 488, catalog no. A-11001, Invitrogen, Waltham, MA). After washing three times with PBS, coverslips were mounted and analyzed using a confocal fluorescence microscope (LSM800, Carl Zeiss).

### 2.10. Experiments on a 3D Model of Human Bronchial Epithelium

After washing the apical side of the epithelia with medium, 10,000PFUs of RSV in a total volume of 100 μL was inoculated apically and incubated for 3 h at 37 °C to allow viral entry. Next, the inoculum was removed with three rapid washing steps, and the residual virus was collected in 200 μL of culture medium added to the apical side for 20 min. Next, 20 μL of compound **1** at 6 µM or 12 µM (or the same amount of DMSO as the control) was added apically to the infected epithelia and then incubated at 37 °C for 24 h to allow for viral replication. Every 24 h, viral progeny was collected apically, as described above, and the treatment was repeated up to 72 h post-infection. Infectious RSV titers of daily-harvested virus were determined with the focus assay. Simultaneously, the toxicity of the treatment was assessed on epithelia using the LDH assay on the basal media derived from the untreated uninfected control, the untreated infected control, and the treated samples collected every 24 h after treatment, as outlined above. The cytotoxicity of the samples was calculated as a percentage, compared to a lysed control tissue sample (100% cytotoxicity). At the end of the experiment, the epithelia were fixed in 4% paraformaldehyde in PBS for 2 h and embedded in paraffin. Samples were then cut (10 µm thick) using a Microtome (Leica, Wetzlar, Germany), and immunofluorescence analysis was carried out to assess tissue morphology after treatment. Briefly, sections from the untreated uninfected control, the untreated infected control, and the treated samples were permeabilized, blocked with PBS containing 0.1% triton X-100 and 10% NGS for 1 h and incubated overnight in a PBS solution with anti-RSV primary antibody (1:100). After primary antibody incubation, the sections were washed three times in PBS and incubated for 1 h in a PBS solution containing the secondary antibody Alexa 488 anti-Mouse (1:200, A-11029, Life Technologies, Carlsbad, CA, USA). The nuclei were stained with 4,6-diamidino-2-phenylindole (DAPI, Sigma-Aldrich, Merck Life Science S.r.l.) diluted 1:1000 in PBS. After three washes in PBS, the sections were mounted with Dako fluorescent mounting medium and analyzed using a Leica SP5 confocal microscope (Leica Microsystems, Wetzlar, Germany).

### 2.11. Statistical Analyses

The results are reported as mean values of three independent experiments performed in duplicate. Values of EC_50_ (half-maximal effective concentration) and CC_50_ (half-maximal cytotoxic concentration) and their respective 95% confidence intervals (CIs) were calculated with regression analysis by fitting a variable slope–sigmoidal dose–response curve. Selectivity indices (SIs) of compounds were calculated from the ratio of CC_50_ to EC_50_. Where suitable, Student’s *t*-test was used to compare virus titers of the treated and untreated samples. Significance is reported for *p* values < 0.05 (*), <0.01 (**), and <0.001 (***).

## 3. Results and Discussion

### 3.1. Investigation of the Antiviral Activity of a PBTZ Library Against Respiratory Viruses

The antiviral potential of the privileged in-house PBTZ library (Table S1) was assayed against the selected respiratory viruses (HCoV-OC43, IFV-A H1N1, RSV-A2) via virus inhibition assays, treating cells with fixed concentrations of compounds (33 μM, 3.3 μM, and 0.33 μM) before, during, and after infection in order to cover all phases of virus replication. Table 1 reports the results of the initial antiviral screening. In total, 10 out of the 29 tested PBTZ analogues strongly inhibited the infectivity of one or more viruses (<20% residual infectivity compared to untreated control). Notably, one-third of the tested PBTZs were highly active against HCoV-OC43, showing <20% infectivity at the highest nontoxic dose. From these data, a clear trend in terms of the structure–activity relationship (SAR) can be observed. Indeed, the contemporary presence of the cyclohexyloxy group at the PBTZ C-8 position, with a free carboxylic function on the moiety linked to the amide at C-4, appears to be crucial in imparting anti-HCoV-OC43 activity (**1**, **2**, **9**, **10**, **14**, **16**, **25**, **27**). In contrast, the PBTZ C-2 substituent appears to exert a relatively minor influence on the anti-HCoV-OC43 activity, with the unsubstituted derivative **27** still demonstrating notable antiviral effects. The same tendency was not observed against RSV and IFV, as fewer PBTZ analogues demonstrated activity without an apparent delineable SAR. Importantly, PBTZ analogue **1** exhibited antiviral activity against all three tested viruses, indicating its potential as a BSAA. Previous studies have already demonstrated the broad-spectrum potential of the PBTZ class. PBTZ analogues were shown to possess anti-flavivirus activity, particularly against Dengue (DENV), Zika, Usutu, West Nile (WNV), Japanese encephalitis, yellow fever, and tick-borne encephalitis viruses [7,8,15,16]. Moreover, Bonotto et al. recently described the anti-SARS-CoV-2 activity of another PBTZ derivative [17].

**Figure 1:**
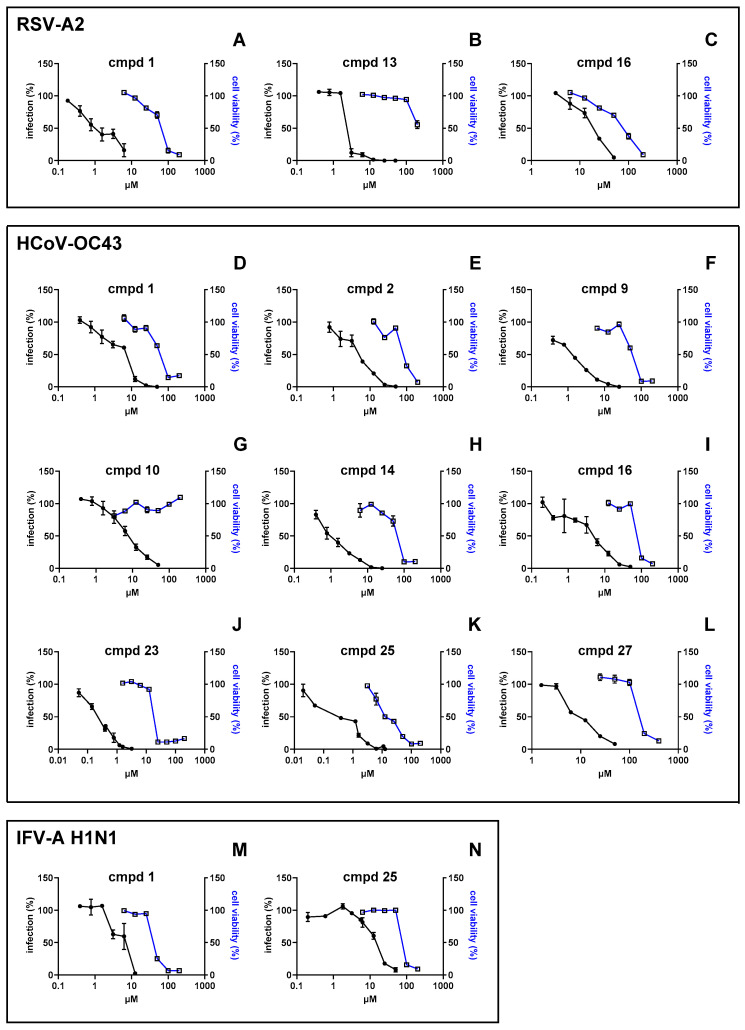
PBTZ analogues exhibited broad-spectrum anti-respiratory virus activity and good cytocompatibility profiles. Antiviral activity of PBTZ was assessed against RSV-A2 (**A**–**C**), HCoV-OC43 (**D**–**L**), and IFV-A H1N1 (**M**,**N**) via the virus inhibition assay by treating cells with serial dilutions of the compound to generate dose–response curves. Viral infectivity (black dots and lines) is reported as the mean percentage of the untreated control ± standard error of the mean (SEM). Cell viability (white squares, blue lines) was calculated as the ratio between the absorbances of treated cells with those of cells incubated with DMSO as the control and expressed as a percentage. Cmpd, compound.

Compounds that showed <20% residual infectivity at the highest nontoxic dose were tested via virus inhibition assays at a wider range of concentrations to generate dose–response curves [12]. Moreover, to exclude the possibility that the observed antiviral activity was due to the cytotoxicity of the compounds, cell proliferation assays were performed. Cell viability was assessed by treating cells under the same experimental conditions as for the corresponding virus inhibition assay. The results of these experiments are depicted in Figure 1, and the EC_50_ and CC_50_ values and selectivity indices (SIs) are reported in detail in Table 2. Interestingly, compound **1** confirmed its broad-spectrum effect, with EC_50_ values in the low µM range against the three respiratory viruses and SIs spanning from 7.49 to 47.2. Other PBTZ analogues demonstrated considerable antiviral efficacy against individual viruses, such as PBTZ derivative **13**, with an EC_50_ of 2.87 µM against RSV (SI > 69.7), and derivative **23**, with an EC_50_ against HCoV-OC43 of 0.23 µM and an SI of 79.1. As internal controls for the experiments, well-characterized and known antiviral compounds were tested against the three viruses, obtaining EC_50_ values similar to those reported in the literature (Table 2) [13,18,19,20].

### 3.2. Focus on the Broad-Spectrum Antiviral Activity of Compound ***1***

Because of its potential as a BSAA, compound **1** was selected for further experimentation. We first confirmed that compound **1** maintained its inhibitory effect on a different cellular model (i.e., the human epithelial lung-derived cell line A549), exhibiting a dose-dependent inhibition of virus infectivity for all viruses tested and EC_50_ values in the same range (Table 3). The slight variation in the EC_50_ values for Hep-2 and A549 cells may be ascribed to a different cellular sensitivity to RSV infection. Indeed, Rajan et al. demonstrated that A549 cells can generate a robust antiviral response against RSV strains compared to Hep-2 cells, with upregulation of IFN-dependent genes and increased cytokine/chemokine expression [21]. A549 cells also showed a higher sensitivity to the compound treatment, compared to the other cell lines used, resulting in greater CC_50_ values. Next, we investigated the compound effect on closely related viral types belonging to the same viral families, testing RSV type B for *Pneumoviridae*, HCoV type 229E for *Coronaviridae*, and IFV type A-H3N2 and IFV type B for *Orthomyxoviridae*. As reported in Table 3, compound **1** was dose-dependently active in inhibiting RSV type B and IFV type A-H3N2 and type B, with EC_50_s similar to those against previously tested viral types. In contrast, HCoV-229E was significantly less inhibited compared to HCoV-OC43, with a ~10-fold higher EC_50_ value. This result could be ascribed to the differences in the viral replicative cycle of these viruses, which differ greatly, especially in the virus attachment and entry steps.

**Figure 2:**
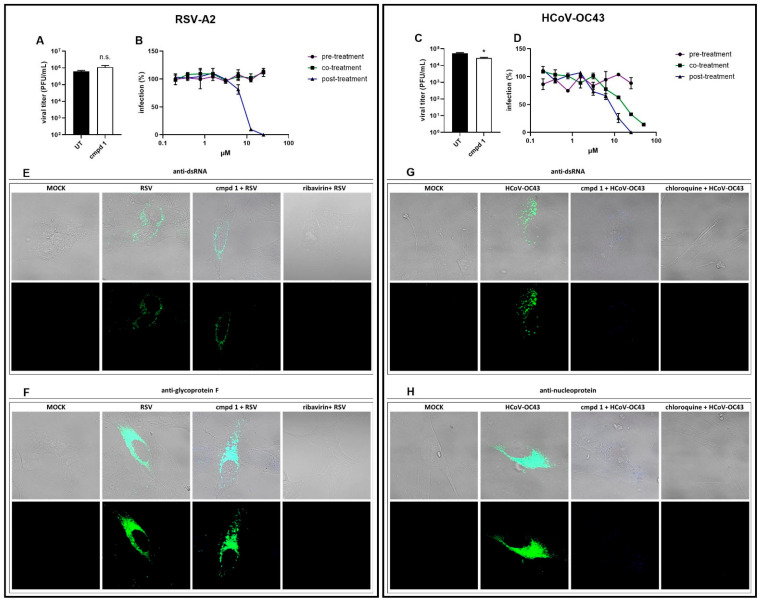
Compound **1** inhibited viral replication at different phases for RSV-A2 and HCoV-OC43. (**A**,**C**) Virucidal assays on infectious RSV-A2 (**A**) and HCoV-OC43 (**C**) particles. Viral titers are expressed as FFU or PFU/mL and are shown as mean ± SEM. UT, untreated. n.s., not significant. *, *p*-value < 0.05. (**B**,**D**) Time-of-addition assays against RSV-A2 (**B**) and HCoV-OC43 (**D**). Viral infectivity is reported as the mean percentage of the untreated control ± SEM. (E-H) Immunofluorescence experiments. Cells were treated with the EC_90_ dose of the compound, ribavirin, and chloroquine and concurrently infected with RSV-A2 (**E**,**F**) or HCoV-OC43 (**G**,**H**). dsRNA (**E**,**G**) and viral proteins (**F**,**H**) are visualized in green, and compound **1** is visualized in blue, with confocal laser microscopy. The pictures are representative of ≥20 images acquired per condition. MOCK, uninfected untreated. Magnification, 630×.

Based on these considerations, we performed specific antiviral assays to explore the antiviral mode of action of compound **1**, focusing on RSV-A2 and HCoV-OC43, the two viruses against which the compound was most active. To test whether the compound exerted virucidal activity, as previously observed for parent compound **2 [17]**, virus inactivation assays were performed by incubating 200,000 PFUs/FFUs of RSV or HCoV with the compound at the EC_90_ dose for 2 h, followed by titration on cells at non-inhibitory concentrations of the compound. The results in Figure 2A show that the compound did not directly inactivate the extracellular RSV particle, while a modest—although negligible—reduction in the HCoV-OC43 titer was detected (Figure 2C). Next, time-of-addition assays were performed, adding the compound to the cells only before, during, or after infection. As reported in Figure 2B, when added to the cells before infection (pre-treatment) or with the virus inoculum in the first 2 h of infection (co-treatment), the compound was not active against RSV, suggesting that early cell–virus interactions were not affected. RSV replication was instead hampered when the infected cells were treated with compound **1** for 72 h after the removal of viral inoculum (post-treatment), with an EC_50_ of 8.2 µM. However, the time-of-addition experiments on HCoV-OC43 showed that the compound was able to inhibit virus replication both during (co-treatment, EC_50_ 16.3 µM) and after the infection (post-treatment, EC_50_ 7.5 µM), whereas antiviral treatment before the infection did not exert inhibitory activity (Figure 2D). These data suggest that compound **1** could act on a viral intracellular replicative step. Therefore, we evaluated the effect of the PBTZ on viral genome replication and viral protein production. Immunofluorescence experiments were conducted on cells treated with the EC_90_ dose of the compound and infected with RSV-A2 or HCoV-OC43. Twenty-four hours post-infection (i.e., after a single cycle of virus replication), the cells were fixed and stained with anti-dsRNA or antiviral protein antibodies. Regarding RSV-A2, no signal reduction in either marker of viral replication could be observed in the compound-treated samples, as the amount of RSV-A2 dsRNA and glycoprotein F in the treated samples was similar to that of the control samples (Figure 2E,F). Analogous results were observed when treating cells with two additional concentrations of compound **1** (EC_50_, 2-fold EC_90_), further strengthening the reliability of the data (Figure S1). Compound **1**, however, clearly inhibited both viral genome replication and the production of the viral nucleoprotein of HCoV-OC43 (Figure 2G,H). As the control, an effective inhibition of both viral RNA replication and viral protein expression was observed when treating cells with two known antiviral drugs, i.e., ribavirin and chloroquine, against RSV-A2 and HCoV-OC43, respectively. Our results suggested that compound **1** may target different steps of the replicative cycle of the two viruses: early replicative steps for HCoV-OC43 occurring before genome replication and late phases of RSV-A2 replication (i.e., virus assembly/egress). The different action of compound **1** observed against HCoV-OC43 and RSV may be ascribed to a distinctive antiviral profile activity of certain PBTZs. Notably, the mechanism of action of PBTZ analogues varies based on chemical functionalization, resulting in the inhibition of flaviviral replication through the direct targeting of NS5 RdRp [8] or the NS3/NS5 interaction [15], as well as the damage to the virion structure [17]. Moreover, a recent work on the anti-DENV activity of certain PBTZ analogues showed that the compounds impeded the capacity of new viral particles to initiate a secondary round of infection, while viral RNA replication was not altered, as we herein demonstrated against RSV [22]. Considering the ability of PBTZ derivatives to alter lipidic membranes, as reported in the literature [17], we can hypothesize that compound **1** is able to interact with the lipidic membranes of cells, inhibiting different phases of viral replication. This exploratory explanation could indeed account for the replicative stage-specific effects seen for RSV and HCoV-OC43. Future studies will focus on the identification of the specific molecular targets involved in PBTZ anti-respiratory virus activity.

**Figure 3:**
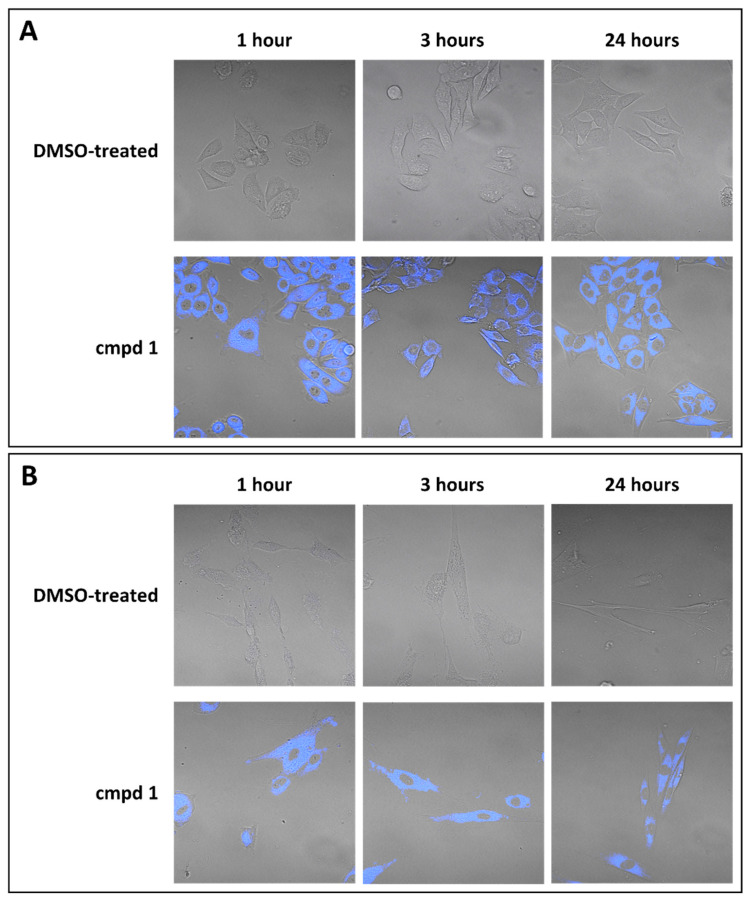
Compound **1** was internalized into host cells. Uninfected Hep2 (**A**) or MRC-5 (**B**) cells were treated with the compound at the corresponding EC_90_ dose for 1 h, 3 h, and 24 h. The uptake of compound **1** in live unfixed cells is visualized in blue with confocal laser microscopy. The control sample (untreated) was incubated with equal amounts of DMSO. Magnification, 400×.

Additionally, since compound **1** presented autofluorescence features conferred by its molecular structure, we analyzed the cellular uptake of the compound in live cells using confocal fluorescence microscopy to assess whether compound **1** was internalized and, thereby, whether it could act intracellularly to inhibit viral replication. Figure 3 shows that compound **1** at the EC_90_ dose was internalized by both cell lines tested (MRC-5 and Hep2 cells) after 1 h of treatment, distributing homogeneously in the cytoplasm. The same distribution was observed after 3 h and 24 h of treatment. These results evidenced, for the first time, the ability of PBTZs, in particular, compound **1**, to penetrate into the cell, highlighting that the chemical properties of the PBTZ enable its passive cellular uptake.

### 3.3. Anti-RSV Activity of Compound ***1*** on a 3D Model of Human Bronchial Epithelium

As a proof of concept, to substantiate its promising antiviral potential, compound **1** was also assayed on an in vitro model that more closely resembles in vivo conditions, i.e., a fully differentiated 3D model of the human bronchial epithelium obtained from a healthy human donor, as previously described [13,23,24]. We selected RSV-A2 to perform these experiments, as compound **1** exerted the strongest inhibitory activity against RSV among the different tested pathogens. Notably, the tissue models employed herein have already been validated for evaluation of the antiviral activity of reference anti-RSV drugs, such as ALS-8112, an anti-RSV nucleoside analogue; GS-5806, a fusion protein inhibitor; and palivizumab, a monoclonal antibody directed against RSV surface protein F [25,26].

**Figure 4:**
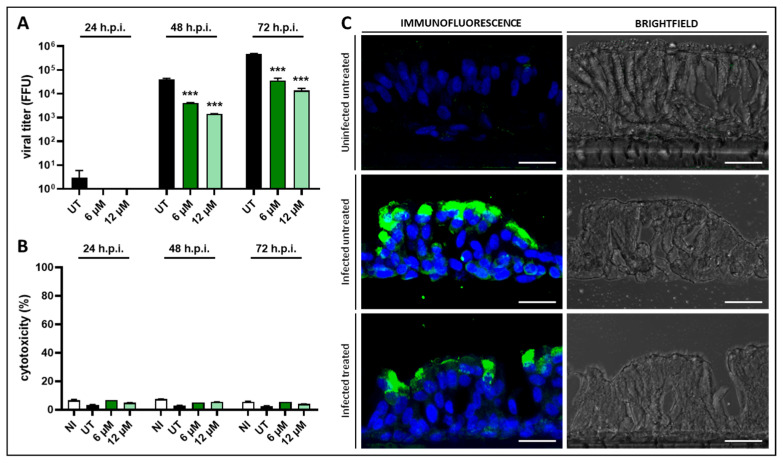
Compound **1** exerted anti-RSV activity on a 3D model of human bronchial epithelium, with no cytotoxic effect. (**A**) Anti-RSV activity of compound **1** on fully reconstructed human bronchial epithelia. Infectious RSV titers of daily collected virus were determined using the focus assay and are reported as total FFU ± SEM for each time point. UT, infected untreated. ***, *p*-value < 0.001. (**B**) Cellular toxicity caused by compound **1** treatment was evaluated with the LDH assay. Cytotoxicity of basal media samples was calculated as a percentage compared to a lysed control tissue sample (100% cytotoxicity). NI, uninfected untreated. UT, infected untreated. (**C**) Morphological analysis of human bronchial epithelia using the immunofluorescence assay. RSV-infected cells, treated with compound **1** or not, are visualized in green (anti-RSV antibody), and cell nuclei are visualized in blue (DAPI), with confocal laser microscopy. Scale bar, 20 µm.

Briefly, 10,000PFUs of RSV were inoculated on the epithelia for 3 h, and compound **1** was administered at fixed doses (6 µM and 12 µM) every 24 h. Over time, RSV production was monitored in terms of harvesting and titrating newly produced viruses daily. The results with 10,000 PFUs of RSV are reported in Figure 4A. Compound **1** inhibited the production of the virus at all time points tested, in a dose-dependent manner. In particular, at 72 h post-infection, the reduction in RSV progeny reached 1.1 and 1.5 log for 6 µM and 12 µM, respectively. In parallel, possible cytotoxic effects of the antiviral treatment on epithelia were monitored via LDH assays on basal media of treated and untreated tissues, harvested daily. Importantly, compound **1** did not exert any cytotoxic effect on the epithelia (Figure 4B).

At the end of the experiment, the epithelia were fixed and subjected to immunofluorescence analysis using an anti-RSV antibody. As shown in Figure 4C, the infected untreated samples showed an intense immunopositive signal, highlighted in green, localized in the apical portion of the tissue section, while compound **1** treatment determined a reduction in immunopositivity, indicating a reduction in virus infection caused by the antiviral treatment. To investigate the organization and morphology of the epithelial cells after infection and drug treatment, brightfield images were acquired. While the control epithelium shows the typical structure of a normal respiratory epithelium characterized by pseudostratified epithelial cells with cilia, in the infected untreated tissue, the pseudostratified arrangement of cells was lost due to viral infection. Importantly, in the infected treated sample, the morphology of the bronchial epithelial cells was sufficiently preserved compared to the infected untreated epithelial cells, indicating protection from virus-induced damage and confirming the good cytocompatibility of the compound.

## 4. Conclusions

Phenotypic screening conducted on a privileged library of PBTZ analogues led to the identification of compound **1**, which is endowed with efficacy against clinically relevant respiratory viruses, including RSV-A2, HCoV-OC43, and IFV-A H1N1. Compound **1** exhibited antiviral activity independent of the viral type and cell type and, notably, maintained its effects in a 3D model of fully differentiated human bronchial epithelium, showing a good cytocompatibility profile. Preliminary mode of action studies suggested that compound **1** inhibits virus infection in different steps of the viral replicative cycle, reminiscent of the polypharmacology observed for other PBTZ derivatives against flaviviruses. Overall, we propose compound **1** as a promising BSAA, although further studies are warranted to reveal the molecular target/s of the compound.

## Figures and Tables

**Table 1:** Screening of the antiviral activity of PBTZ analogues.

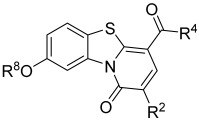												
Cmpd	R8	R2	R4	Residual Virus Infectivity (%)								
				RSV-A2	HCoV-OC43			IFV-A H1N1				
				33 µM	3 µM	0.3 µM	33 µM	3 µM	0.3 µM	33 µM	3 µM	0.3 µM
**1**	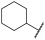	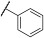	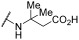	- ^a^	20	38	0	73	100	2	56	100
**2**	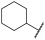	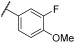	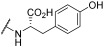	-	44	100	1	54	95	100	100	100
**3**	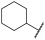	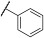	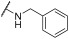	67	93	100	68	75	83	78	100	100
**4**	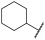	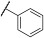	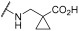	-	57	63	-	55	74	42	100	100
**5**	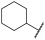 in C-7	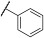	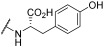	-	71	73	-	81	88	100	100	100
**6**	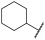	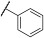	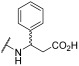	-	-	76	-	84	87	38	100	100
**7**	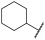	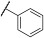	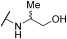	-	-	80	-	63	57	-	83	100
**8**	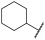	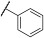	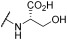	98	100	100	47	88	100	94	100	100
**9**	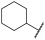	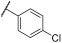	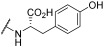	-	89	96	0	27	81	50	98	100
**10**	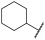	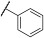	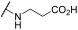	-	85	100	12	75	98	100	100	100
**11**	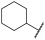	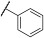	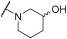	-	-	62	-	43	100	-	49	100
**12**	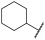	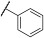	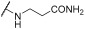	-	91	96	94	97	100	62	93	100
**13**	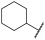	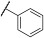	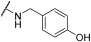	0	15	100	64	100	100	62	100	100
**14**	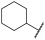	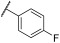	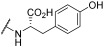	-	99	100	0	22	85	84	100	100
**15**	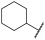	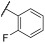	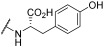	40	85	86	41	70	81	100	100	100
**16**	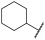	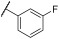	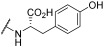	19	100	100	5	70	87	100	100	100
**17**	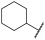	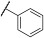	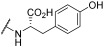	-	51	81	40	67	100	100	100	100
**18**	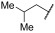	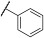	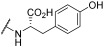	42	100	100	26	72	94	100	100	100
**19**	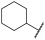	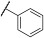	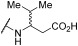	-	22	73	-	48	92	-	45	100
**20**	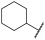	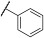	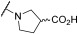	100	100	100	41	59	100	100	100	100
**21**	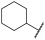	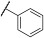	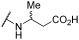	-	56	100	-	49	76	-	100	100
**22**	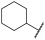	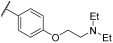	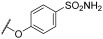	-	68	99		40	69	-	100	100
**23**	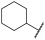	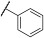	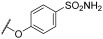	-	100	100	81	100	100	52	73	100
**24**	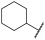	-H	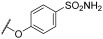	-	64	75	-	67	100	-	44	100
**25**	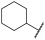	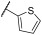	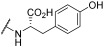	-	85	89	0	7	49	18	85	89
**26**	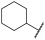	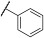	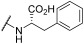	-	53	100	-	64	79	83	100	100
**27**	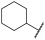	-H	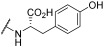	100	100	100	17	92	100	81	99	100
**28**	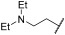	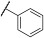	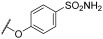	-	97	100	-	0	32	-	83	100
**29**	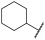	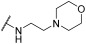	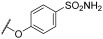	-	67	100	-	100	100	-	100	100

^a^ Cytotoxic effect microscopically visible; Cmpd: compound.

**Table 2:** Antiviral activity and cytotoxicity of selected compounds against RSV, HCoV, and IFV.

Virus	Compound	EC_50_^a^ (µM) (95% CI ^b^)	CC_50_^c^ (µM) (95% CI)	SI ^d^
RSV-A2	**1**	1.30 (0.87–1.98)	61.3 (52.3–71.3)	47.2
	**13**	2.87 (2.13–3.67)	>200	>69.7
	**16**	18.6 (15.9–21.8)	72.9 (64.2–82.8)	3.91
	ribavirin	37.0 (24.8–52.9)	>2400	>64.9
HCoV-OC43	**1**	4.83 (3.92–5.29)	59.6 (49.5–72.0)	12.3
	**2**	4.67 (3.69–5.87)	82.9 (75.1–98.2)	17.8
	**9**	1.20 (1.04–1.37)	55.6 (47.9–65.1)	46.4
	**10**	7.97 (6.47–9.79)	>200	>25.1
	**14**	1.09 (0.89–1.33)	62.2 (51.7–74.2)	57.1
	**16**	4.22 (2.82–6.21)	84.7 (71.2–97.8)	20.1
	**23**	0.23 (0.20–0.26)	18.2 (12.7–23.2)	79.1
	**25**	0.29 (0.18–0.47)	16.5 (13.5–20.3)	57.0
	**27**	10.0 (8.29–12.2)	188.9 (154.2–223.0)	18.9
	chloroquine	0.19 (0.13–0.26)	169.3 (150.4–190.5)	891.1
IFV-A H1N1	**1**	5.49 (3.85–7.83)	41.1 (37.8–47.1)	7.49
	**25**	13.7 (11.8–15.7)	86.1 (74.7–90.2)	6.28
	oseltamivir	0.17 (0.04–0.42)	>200	>1176

^a^ EC_50_: half-maximal effective concentration; ^b^ CI: 95% confidence interval; ^c^ CC_50_: half-maximal cytotoxic concentration; ^d^ SI: selectivity index.

**Table 3:** Anti-respiratory virus activity of compound **1** against different viral strains and on different cell lines.

Virus	Strain	Cell Line	EC_50_^a^ (µM) (95% CI ^b^)	CC_50_^c^ (µM) (95% CI)	SI ^d^
RSV	A2	Hep-2	1.30 (0.87–1.98)	61.3 (52.3–71.3)	47.2
		A549	7.78 (6.92–9.10)	30.8 (27.6–34.4)	3.96
	B WV/14617/85	Hep-2	4.23 (3.19–5.63)	61.3 (52.3–71.3)	14.5
HCoV	OC43	MRC-5	4.83 (3.92–5.29)	59.6 (49.5–72.0)	12.3
		A549	8.14 (7.22–9.34)	150.2 (142.0–158.9)	18.5
	229E	MRC-5	45.3 (37.3–49.2)	59.6 (49.5–72.0)	1.32
IFV	A-H1N1	MDCK	5.49 (3.85–7.83)	41.1 (37.8–47.1)	7.49
		A549	6.29 (4.50–8.42)	57.6 (51.5–64.6)	9.16
	A-H3N2	MDCK	5.96 (4.66–7.82)	41.1 (37.8–47.1)	6.90
	B	MDCK	4.21 (3.40–5.57)	41.1 (37.8–47.1)	9.76

^a^ EC_50_: half-maximal effective concentration; ^b^ CI: 95% confidence interval; ^c^ CC_50_: half-maximal cytotoxic concentration; ^d^ SI: selectivity index.

## Data Availability

The original contributions presented in this study are included in this article/the Supplementary Materials. Further inquiries can be directed to the corresponding author(s).

## Supplementary Materials

The following supporting information can be downloaded at https://www.mdpi.com/article/10.3390/v17070890/s1, Supplementary Materials and Methods; Table S1: Chemical structures of the tested compounds. Figure S1: Compound **1** at different doses inhibited viral replication at different phases for RSV-A2.

## Abbreviations

The following abbreviations are used in this manuscript:

HCoV	human coronavirus
RSV	respiratory syncytial virus
IFV	influenza virus
PBTZ	pyridobenzothiazolone
cmpd	compound
EC_50_	50% effective concentration
EC_90_	90% effective concentration
CC_50_	Half-maximal cytotoxic concentration
SI	selectivity index
PFU	plaque-forming unit
PFU/mL	plaque-forming unit per mL
FFU	focus-forming unit
FFU/mL	focus-forming unit per mL
