# Supplementary material for: Broad-Spectrum Antiviral Activity of Pyridobenzothiazolone Analogues Against Respiratory Viruses
Source: Viruses. 2025 Jun 24;17(7):890. doi: 10.3390/v17070890 (PMC12299477; doi:10.3390/v17070890)
Supplement: Supplementary file 1 [file viruses-17-00890-s001.zip › viruses-3632382-supplementary.pdf]

Broad-Spectrum Antiviral Activity of Pyridobenzothiazolone Analogues Against Respiratory Viruses

Elisa Feyles [1,†], Tommaso Felicetti [2,†], Irene Arduino [1], Massimo Rittà [1], Andrea Civra [1], Luisa Muratori [3], Stefania Raimondo [3], David Lembo [1], Giuseppe Manfroni [2,‡] and Manuela Donalisio [1,*,‡]

1 Laboratory of Molecular Virology and Antiviral Research, Department of Clinical and Biological Sciences, University of Turin, Regione Gonzole 10, 10043 Orbassano, Italy; elisa.feyles@unito.it (E.F.); irene.arduino@unito.it (I.A.); massimo.ritta@unito.it (M.R.); andrea.civra@unito.it (A.C.); david.lembo@unito.it (D.L.)

2 Department of Pharmaceutical Sciences, University of Perugia, Via del Liceo, 1, 06123 Perugia, Italy; tommaso.felicetti@unipg.it (T.F.); giuseppe.manfroni@unipg.it (G.M.)

3 Department of Clinical and Biological Sciences, Neuroscience Institute Cavalieri Ottolenghi (NICO), University of Turin, Regione Gonzole 10, 10043 Orbassano, Italy; luisa.muratori@unito.it (L.M.); stefania.raimondo@unito.it (S.R.)

* Correspondence: manuela.donalisio@unito.it; Tel.: +39-011-6705427

† These authors contributed equally to this work.

‡ These authors share senior authorship.

Supplementary Materials and Data

Materials and Methods

Chemistry

The synthesis of the tested compounds **1-21** has been previously reported and the appropriate reference for each compound has been added in **Table S1**. In contrast, the synthetic procedures of the tested compounds **22-29** (which are part of the in-house library but have not been previously reported) are depicted below in the Schemes 1 and 2.

The synthesis of compounds **22-27** is illustrated in **Scheme 1**. The pyridobenzothiazolone ester derivatives **33** and **37** were obtained through a reaction between the acrylate intermediate **30**, which was prepared accordingly to the previously established procedure[1], and the anhydride **32** or the 2-thienylacetic anhydride, respectively, at 110 °C in neat conditions. It is noteworthy that anhydride **32** was freshly prepared by reacting the (4-{[*tert*-butyl(dimethyl)silyl]oxy}phenyl)acetic acid in the presence of DCC in dry toluene. The deprotection of **33** with Cs_2CO_3 in a mixture of dry DMF and H_2O yielded the phenol derivative **34**, which was subsequently reacted with the 2-(diethylamino)ethyl chloride hydrochloride using K_2CO_3 as a base in dry DMF, affording compound **35**. At this stage, the basic hydrolysis of ester analogues **35** and **37** and of ester compounds **38** and **39**, which were prepared accordingly to the previously established procedures[1,2], was conducted using a mixture of aqueous 10% NaOH solution and MeOH. This afforded acid analogues **36**, **40**, **41**[1] and **42**[2]. Subsequently, the reaction of compounds **36**, **41**[1] and **42**[2] with 4-hydroxybenzene sulfonamide using BOP and DIPEA or Et_3N in dry DMSO yielded the target compounds **22-24**. Concurrently, the reactions of acid derivatives **40** and **42**[2] with tyrosine methyl ester hydrochloride and of acid derivative **41**[1] with phenylalanine methyl ester hydrochloride, in the presence of TBTU and DIPEA in dry DMSO, resulted in the formation of ester amino acid analogues **43**, **45** and **44**, respectively. Subsequent mild basic hydrolysis using a mixture of 1N LiOH in H_2O and dioxane yielded the target compounds **25**, **27** and **26**, respectively.

Scheme S1: synthesis of compounds **22-27**.

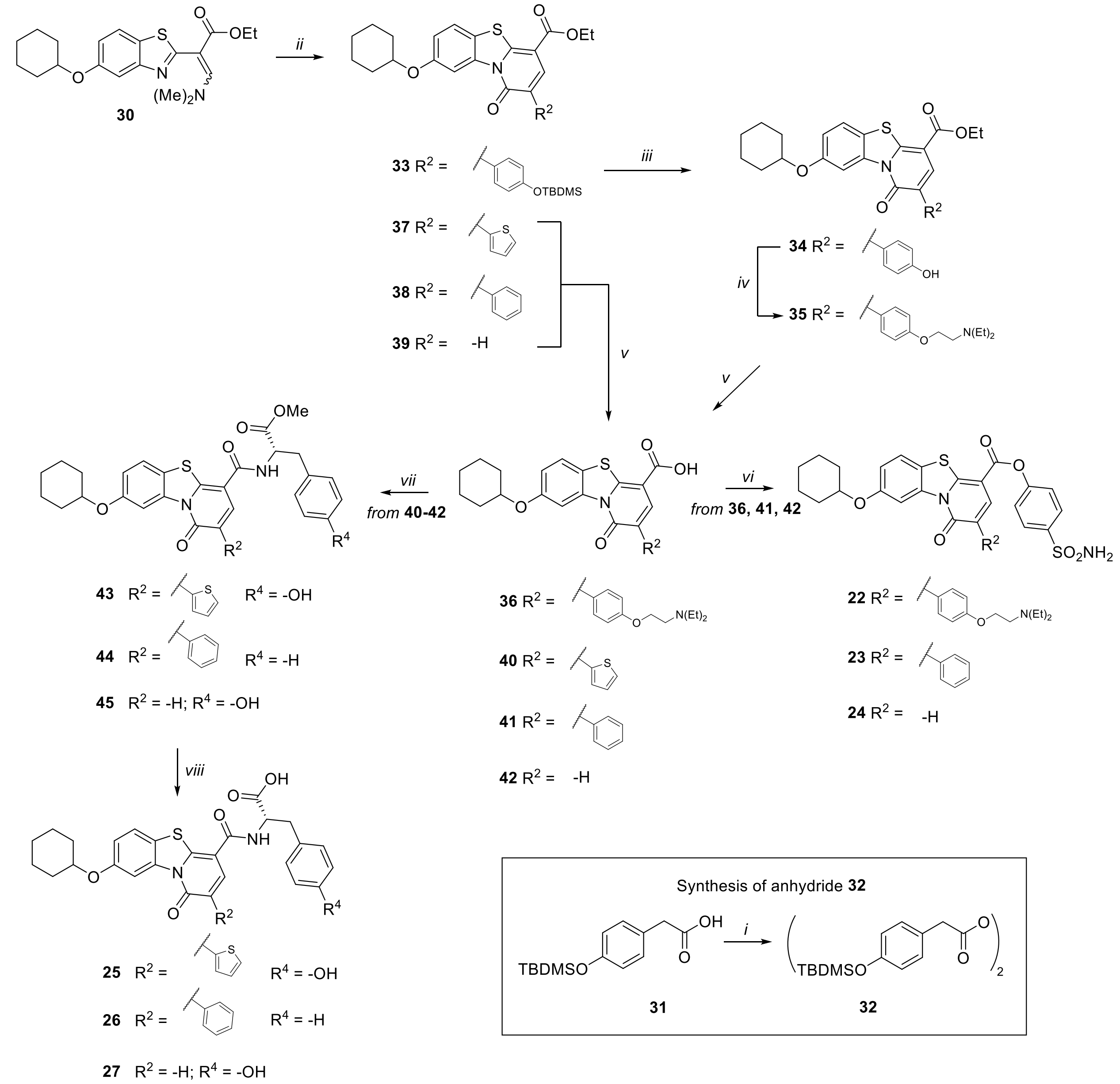

Reagents and conditions: i) DCC, dry toluene, rt, 12h; ii) **32** or 2-thienylacetic anhydride, neat, 110 °C, 1-5h; iii) Cs_2CO_3, DMF:H_2O (10:1), rt, 12h; iv) 2-(diethylamino)ethyl chloride hydrochloride, K_2CO_3, dry DMF, rt, 3h; v) 10% aq. NaOH, MeOH, 80 °C, 2-3h; vi) 4-hydroxybenzenesulfonamide, BOP, DIPEA or Et_3N, dry DMSO, rt, 1h, vii) tyrosine methyl ester hydrochloride or phenylalanine methyl ester hydrochloride, TBTU, DIPEA, dry DMSO, r.t., 1.5-4h; viii) aq. 1N LiOH, dioxane, r.t., 1-4h.

The synthesis of compounds **28** and **29** is depicted in **Scheme 2**. The cyclohexyl moiety of pyridobenzothiazolone ester derivative **38**, which was prepared in accordance with the previously established procedure[1], was removed with 1M BBr_3 in CH_2Cl_2 to afford the hydroxy derivative **46**. Subsequently, the alkylation of **46** with 2-(diethylamino)ethyl chloride hydrochloride, in the presence of K_2CO_3 in dry DMF, yielded derivative **47**. Concurrently, the selective bromination of the ester derivative **39**, which was prepared in accordance with the previously established procedure[2], with Br_2 in AcOH afforded the C-2 bromo derivative **49**, which was subsequently employed in a Buchwald-Hartwig Pd-catalyzed amination with 4-(2-aminoethyl)morpholine in the presence of

BINAP, Pd(OAc)2 and NaO*t*Bu in dry toluene, affording derivative **50**. The basic hydrolysis of compounds **47** and **50** in a mixture of aqueous 10% NaOH in H_2O and MeOH at 80 °C afforded the corresponding acid derivatives **48** and **51**. These were then reacted with 4-hydroxybenzene sulfonamide, in the presence of BOP and Et_3N, in dry DMSO, resulting in the formation of target compounds **28** and **29**.

Scheme S2: synthesis of compounds **28** and **29**.

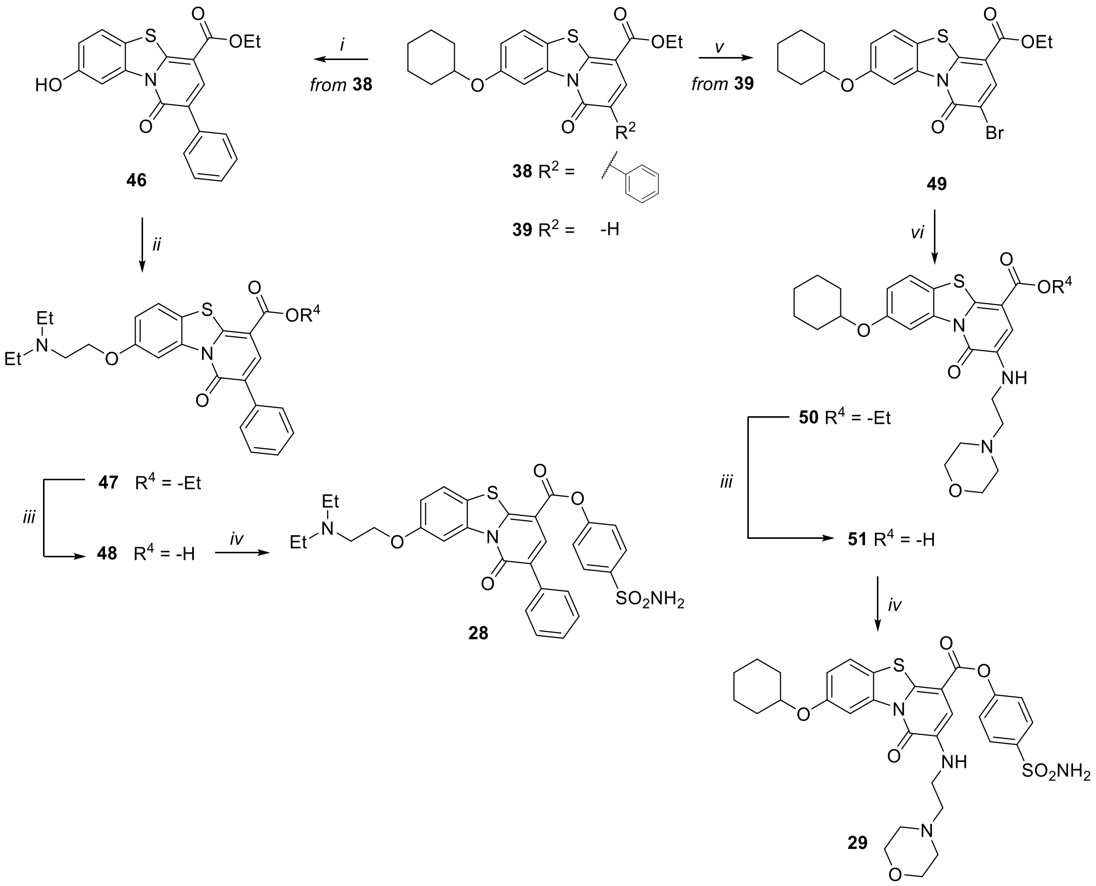

Reagents and conditions: i) 1M BBr_3 in CH_2Cl_2, dry CH_2Cl_2, 0 °C, 1h; ii) 2-(diethylamino)ethyl chloride hydrochloride, K_2CO_3, dry DMF, 80 °C, 2h; iii) 10% aq. NaOH, MeOH, 80 °C, 2h; iv) 4-hydroxybenzene sulfonamide, BOP, Et_3N, dry DMSO, r.t., 1-3h; v) Br_2, AcOH, rt, 30 min.; vi) 4-(2-aminoethyl)morpholine, BINAP, $Pd(OAc)_2$, NaO*t*Bu, dry toluene, reflux, 12h.

General chemistry

Unless otherwise stated, all starting materials were procured from commercial sources. All reagents and solvents were procured from standard commercial suppliers and were utilized without modification. The organic solutions were dried using anhydrous Na_2SO_4 and concentrated with a rotary evaporator (IKA RV 3V) under reduced pressure, with a VACSTAR digital IKA pump employed for this purpose. All reactions were routinely checked by TLC on silica gel 60_{F254} (Merck), with visualization conducted through the use of ultraviolet (UV) radiation and iodine. Purifications were conducted via flash column chromatography separations using Merck silica gel 60 (mesh 230–400). The yield data provided herein refer to the purified products and are not optimized. The ^1H and ^{13}C NMR spectra were recorded at 400 and 100 MHz, respectively, using a Bruker Advance DRX-400 instrument. The chemical shifts (δ) are reported in parts per million (ppm) relative to tetramethylsilane (TMS) and calibrated using residual undeuterated solvent as the internal reference. The values of the coupling constants (*J*) are expressed in hertz (Hz). The spectra were acquired at 298

K, and the data were processed using Bruker TopSpin 4.4.0 software. The resulting spectral data are consistent with the assigned structures. The purity (>95%) of all compounds, both those synthesized and those from the in-house library, was evaluated by HPLC analysis, employing a Jasco LC-4000 instrument equipped with a UV-Visible Diode Array Jasco MD-4015. For Methods A and B, a Gemini LC C18 110 Å column, 3 µm, 100 mm × 2 mm (Phenomenex) was employed with a flow rate of 0.5 mL/min. Method A: the acquisition time was of 12 min, during which a gradient of CH_3CN and H_2O containing 0.1% of formic acid was employed (20 to 100% CH_3CN over 10 min, followed by 2 min with 100% CH_3CN). Method B: the acquisition time was of 15 min, during which a gradient of CH_3CN and H_2O containing 0.1% of formic acid was employed (50 to 100% CH_3CN over 15 min). For Method C, an XTerra MS C18 Column, 5 µm, 4.6 mm × 150 mm (Waters Corporation) was employed with a flow rate of 0.6 mL/min. The acquisition time was of 12 min, during which a gradient of CH_3CN and H_2O containing 0.1% of diethylamine was employed (10 to 100% CH_3CN over 10 min, followed by 2 min with 100% CH_3CN).
The peak retention time is indicated in minutes. The HRMS detection was based on electrospray ionization (ESI) in negative or positive polarity, using an Agilent UHPLC 1290 Infinity II System equipped with an Agilent 6560 QTOF Ion Mobility MS detector.

Experimental procedures for the synthesis of target compounds

General procedure A. Under N_2 atmosphere, to a solution of ester analogue (1 equiv.) in dry DMF (3 mL per mmol), K_2CO_3 (3 equiv.) and the appropriate electrophile (2 equiv.) were added, and the reaction was stirred for the specified duration at the designated temperature. Subsequently, the mixture was poured into ice/water, and the precipitate was filtered under vacuum to afford the desired compounds as solids, which were used as such for the next reactions.

General procedure B. To a solution of ester analogue (1 equiv.) in MeOH (15 mL per mmol), 10% NaOH in H_2O (7.5 mL per mmol) was added, and the reaction was stirred at 80 °C for the specified duration. Subsequently, the mixture was poured into ice/water, the pH was adjusted to 5 with 2N HCl and the precipitate was filtered under vacuum to afford the desired compounds as solids, which were used as such for the next reactions.

General procedure C. Under N_2 atmosphere, to a solution of acidic analogue (1 equiv.) in dry DMSO (3 mL per mmol), BOP or TBTU (1.5 equiv.), Et_3N or DIPEA (5 equiv.) and the appropriate phenol or amino acid methyl ester hydrochloride (2 equiv.) were added, and the reaction was stirred at rt for the specified duration. Subsequently, the mixture was poured into ice/water and acidified with 2N HCl (pH = 4). Upon the formation of a precipitate, subsequent filtration under vacuum yielded the desired compounds in a solid state. For **45**, the mixture underwent three extractions with EtOAc, after which the organic layer was washed with brine, dried over Na_2SO_4, and evaporated to dryness. The crude solids were purified or utilized as such, as indicated for each compound.

General procedure D. A solution of the appropriate methyl ester (1 equiv.) and aq. 1N LiOH (5 equiv.) in 1,4-dioxane (10 mL) was stirred at room temperature (1-4h). The mixture was poured into ice/water, acidified with 2N HCI (pH 3) and extracted three times with EtOAc. The combined organic layers were washed two times with brine, dried, and evaporate under vacuum to give a yellow solid which was purified as indicated for each compound.

(4-{[*tert*-butyl(dimethyl)silyl]oxy}phenyl)acetic anhydride (32). Under N_2 atmosphere, to a solution of (4-{[*tert*-butyl(dimethyl)silyl]oxy}phenyl)acetic acid (**31**) (8.60 g, 32.28 mmol) in dry toluene (60 mL), DCC (3.73 g, 18.08 mmol) was added portionwise and the reaction mixture was stirred at rt for 12h. Subsequently, the mixture was filtered, and the filtrate was evaporated to dryness to afford a yellow oil in 78% yield (12.96 g), which was used as such for the next reaction. ^{1}H NMR (400 MHz, $CDCl_3$): δ = 7.01-6.98 (m, 4H, Ar-H), 6.77-6.72 (m, 4H, Ar-H), 3.61 (s, 4H, CH_2 x2), 0.95 (s, 18H, $C(CH_3)_3$ x2), 0.15 (s, 12H, $Si(CH_3)_2$ x2).

Ethyl 2-(4-{[*tert*-butyl(dimethyl)silyl]oxy}phenyl)-8-(cyclohexyloxy)-1-oxo-1*H*-pyrido[2,1-*b*][1,3]benzothiazole-4-carboxylate (33). A mixture of the acrylate intermediate **30**[1] (0.43 g, 1.14 mmol) and the freshly prepared anhydride **32** (1.76 g, 3.42 mmol) was stirred in a 110 °C pre-heated oil bath for 5h. After allowing the mixture to cool to rt, MeOH (15 mL) was added and the precipitate was filtered to afford derivative **55** as a yellow solid in 62% yield. ^{1}H NMR (400 MHz, $CDCl_3$): δ = 9.11 (d, *J* = 2.4 Hz, 1H, Ar-H), 8.19 (s, 1H, Ar-H), 7.69-7.62 (m, 3H, Ar-H), 7.19 (dd, *J* = 2.4 and 8.6 Hz, 1H, Ar-H), 6.97-6.92 (m, 2H, Ar-H), 4.49-4.41 (m, 3H, OCH and O$CH_2$$CH_3$), 2.03-1.95 (m, 2H, cyclohexyl-CH_2), 1.81-1.77 (m, 2H, cyclohexyl-CH_2), 1.68-1.55 (m, 4H, cyclohexyl-CH_2 x2), 1.49-1.39 (m, 5H, cyclohexyl-CH_2 and $OCH_2$$CH_3$), 1.05 (s, 9H, $C(CH_3)_3$), 0.25 (s, 6H, $Si(CH_3)_2$).

Ethyl 8-(cyclohexyloxy)-2-(4-hydroxyphenyl)-1-oxo-1*H*-pyrido[2,1-*b*][1,3]benzothiazole-4-carboxylate (34). To a suspension of the derivative **33** (0.31 g, 0.54 mmol) in a mixture of DMF/H_2O (10:1 – 10 mL), Cs_2CO_3 (0.09 g, 0.27 mmol) was added portionwise and the reaction was stirred at rt for 12h. Subsequently, H_2O was added, the pH was adjusted to 5 by the addition of 2N HCl, and the precipitate was filtered to give compound **34** as a yellow solid in 95% yield. ^{1}H NMR (400 MHz, DMSO-*d6*): δ = 9.31 (s, 1H, OH), 8.80 (s, 1H, Ar-H), 7.95 (s, 1H, Ar-H), 7.89 (d, *J* = 8.8 Hz, 1H, Ar-H), 7.45 (d, *J* = 8.4 Hz, 2H, Ar-H), 7.17 (d, *J* = 9.0 Hz, 1H, Ar-H), 6.71 (d, *J* = 8.5 Hz, 2H, Ar-H), 4.38-4.27 (m, 3H, OCH and O$CH_2$$CH_3$), 1.95-1.88 (m, 2H, cyclohexyl-CH_2), 1.73-1.65 (m, 2H, cyclohexyl-CH_2), 1.55-1.47 (m, 4H, cyclohexyl-CH_2 x2), 1.42-1.29 (m, 5H, cyclohexyl-CH_2 and $OCH_2$$CH_3$).

Ethyl 8-(cyclohexyloxy)-2-{4-[2-(diethylamino)ethoxy]phenyl}-1-oxo-1*H*-pyrido[2,1-*b*][1,3]benzothiazole-4-carboxylate (35). Following the general procedure A, starting from compound **34** and using 2-(diethylamino)ethyl chloride hydrochloride as electrophile (temperature: rt; time: 3h), compound **35** was obtained as a yellow solid in 68% yield (0.23 g). ^{1}H NMR (400 MHz, $CDCl_3$): δ = 9.09 (d, *J* = 2.4 Hz, 1H, Ar-H), 8.21 (s, 1H, Ar-H), 7.71-7.68 (m, 2H, Ar-H), 7.62 (d, *J* = 8.7 Hz, 1H, Ar-H), 7.16 (dd, *J* = 2.4 and 8.7 Hz, 1H, Ar-H), 7.07-7.01 (m, 2H, Ar-H), 4.49-4.45 (m, 3H, OCH and O$CH_2$$CH_3$), 4.19 (t, *J* = 6.4 Hz, 2H, O$CH_2$$CH_2$N), 2.95 (t, *J* = 6.3 Hz, 2H, $OCH_2$$CH_2$N), 2.69 (q, *J* = 6.1 Hz, 4H, N$CH_2$$CH_3$ x2), 2.07-1.99 (m, 2H, cyclohexyl-CH_2), 1.81-1.75 (m, 2H, cyclohexyl-CH_2), 1.63-1.42 (m, 6H, cyclohexyl-CH_2 x3), 1.48 (t, *J* = 6.4 Hz, 3H, $OCH_2$$CH_3$), 1.09 (t, *J* = 7.1 Hz, 6H, $NCH_2$$CH_3$ x2).

8-(Cyclohexyloxy)-2-{4-[2-(diethylamino)ethoxy]phenyl}-1-oxo-1*H*-pyrido[2,1-*b*][1,3]benzothiazole-4-carboxylic acid (36). Following the general procedure B, starting from compound **35** (time: 3h), compound **36** was obtained as a yellow solid in 60% yield (0.38 g). ^{1}H NMR (400 MHz, $CDCl_3$): δ = 11.99 (bs, 1H, CO_2H), 8.87-8.81 (m, 1H, Ar-H), 8.13 (s, 1H, Ar-H), 7.96 (d, *J* = 8.6 Hz, 1H, Ar-H), 7.75-7.71 (d, *J* = 7.2 Hz, 2H, Ar-H), 7.25 (d, *J* = 7.1 Hz, 1H, Ar-H), 7.07 (d, *J* = 7.2 Hz, 2H, Ar-H), 4.47-4.41 (m, 3H, OCH and O$CH_2$$CH_3$), 3.50-3.42 (m, 2H, O$CH_2$$CH_2$N), 3.23-3.18 (m, 6H, $OCH_2$$CH_2$N and N$CH_2$$CH_3$ x2), 1.99-1.95 (m, 2H, cyclohexyl-CH_2), 1.76-1.69 (m, 2H, cyclohexyl-CH_2), 1.55-1.47 (m, 4H, cyclohexyl-CH_2 x2), 1.41-1.36 (m, 2H, cyclohexyl-CH_2), 1.27-1.23 (m, 6H, $NCH_2$$CH_3$ x2).

4-(Aminosulfonyl)phenyl 8-(cyclohexyloxy)-2-{4-[2-(diethylamino)ethoxy]phenyl}-1-oxo-1*H*-pyrido[2,1-*b*][1,3]benzothiazole-4-carboxylate (22). Following the general procedure C, starting from compound **36** and using 4-hydroxybenzenesulfonamide, BOP and Et_3N (time: 1h), compound **22** was obtained after filtration and purification by flash chromatography column eluting with $CHCl_3$/MeOH 93:7 as a yellow solid in 65% yield (0.25 g). ^{1}H NMR (400 MHz, DMSO-*d6*): δ = 8.86-8.83 (m, 1H, Ar-H), 8.27 (s, 1H, Ar-H), 8.03 (d, *J* = 8.2 Hz, 1H, Ar-H), 7.95 (d, *J* = 8.2 Hz, 2H, Ar-H), 7.74 (d, *J* = 8.2 Hz, 2H, Ar-H), 7.56 (d, *J* = 8.2 Hz, 2H, Ar-H), 7.47 (bs, 2H, SO_2NH_2), 7.28-7.23 (m, 1H, Ar-H), 7.04 (d, *J* = 8.5 Hz, 2H, Ar-H), 4.44-4.36 (m, 1H, OCH), 4.12-4.06 (m, 2H, O$CH_2$$CH_2$N), 2.87-2.80 (m, 2H, $OCH_2$$CH_2$N), 2.65-2.55 (m, 4H, N$CH_2$$CH_3$ x2), 2.01-1.91 (m, 2H, cyclohexyl-CH_2), 1.78-1.68 (m, 2H, cyclohexyl-CH_2), 1.58-1.27 (m, 6H, cyclohexyl-CH_2 x2), 1.01 (t, *J* = 6.9 Hz, 6H, $OCH_2$$CH_3$ x2). ^{13}C NMR (100 MHz, DMSO-*d6*): δ = 163.09, 161.61, 158.68,

156.88, 154.78, 153.21, 142.33, 139.37, 134.85, 130.51, 128.50, 127.95, 123.83, 123.20, 123.17, 119.45, 116.95, 114.72, 107.37, 101.40, 75.80, 51.84, 47.55 (2C), 31.63, 25.62, 23.57, 12.32. HPLC, Method A: r_t = 6.40 min. HRMS calculated for $C_{36}H_{39}N_3O_7S_2$ $[M+H]^+$ 690.2302, found 690.23247.

4-(Aminosulfonyl)phenyl 8-(cyclohexyloxy)-1-oxo-2-phenyl-1*H*-pyrido[2,1-*b*][1,3]benzothiazole-4-carboxylate (23). Following the general procedure C, starting from compound **41** and using 4-hydroxybenzenesulfonamide, BOP and Et_3N (time: 1h), compound **23** was obtained after filtration and purification by flash chromatography column eluting with CH_2Cl_2/acetone 95:5 as a yellow solid in 61% yield (0.15 g). ^{1}H NMR (400 MHz, DMSO-d_6): δ = 8.86-8.79 (m, 1H, Ar-H), 8.34-8.29 (m, 1H, Ar-H), 8.06-7.99 (m, 1H, Ar-H), 7.95 (d, J = 8.7 Hz, 2H, Ar-H), 7.80 (d, J = 8.1 Hz, 2H, Ar-H), 7.56 (d, J = 8.6 Hz, 2H, Ar-H), 7.53-7.44 (m, 4H, Ar-H and SO_2NH_2), 7.43-7.38 (m, 1H, Ar-H), 7.29-7.22 (m, 1H, Ar-H), 4.45-4.34 (m, 1H, OCH), 2.01-1.92 (m, 2H, cyclohexyl-CH_2), 1.78-1.68 (m, 2H, cyclohexyl-CH_2), 1.56-1.25 (m, 6H, cyclohexyl-CH_2 x3). ^{13}C NMR (100 MHz, DMSO-d_6): δ = 162.92, 161.38, 156.85, 155.33, 153.13, 142.27, 139.23, 136.29, 135.76, 129.25, 128.67, 128.16, 127.87, 123.72, 123.28, 123.07, 119.35, 116.96, 107.12, 101.36, 75.78, 31.55, 25.51, 23.49. HPLC, Method B: r_t = 9.51 min. HRMS calculated for $C_{30}H_{26}N_2O_6S_2$ $[M+H]^+$ 575.1305, found 575.13139.

4-(Aminosulfonyl)phenyl 8-(cyclohexyloxy)-1-oxo-1*H*-pyrido[2,1-*b*][1,3]benzothiazole-4-carboxylate (24). Following the general procedure C, starting from compound **42** and using 4-hydroxybenzenesulfonamide, BOP and DIPEA (time: 1h), compound **24** was obtained after filtration and purification by flash chromatography column eluting with $CHCl_3$/MeOH 99:1 as a yellow solid in 17% yield (0.05 g). ^{1}H NMR (400 MHz, DMSO-d_6): δ = 8.78 (d, J = 2.0 Hz, 1H, Ar-H), 8.26 (d, J = 9.6 Hz, 1H, Ar-H), 8.02 (d, J = 8.8 Hz, 1H, Ar-H), 7.94 (d, J = 8.5 Hz, 2H, Ar-H), 7.53 (d, J = 8.6 Hz, 2H, Ar-H), 7.47 (bs, 2H, SO_2NH_2), 7.26 (dd, J = 2.1 and 9.0 Hz, 1H, Ar-H), 6.47 (d, J = 9.6 Hz, 1H, Ar-H), 4.46-4.39 (m, 1H, OCH), 2.02-1.94 (m, 2H, cyclohexyl-CH_2), 1.83-1.70 (m, 2H, cyclohexyl-CH_2), 1.61-1.27 (m, 6H, cyclohexyl-CH_2 x2). ^{13}C NMR (100 MHz, DMSO-d_6): δ = 162.78, 162.03, 156.78, 156.72, 153.03, 142.17, 138.92, 137.95, 127.78, 123.55, 122.97, 118.96, 116.51, 112.39, 107.06, 101.23, 75.76, 31.48, 25.43, 23.46. HPLC, Method B: r_t = 5.74 min. HRMS calculated for $C_{24}H_{22}N_2O_6S_2$ $[M+H]^+$ 499.0992, found 499.09952.

Ethyl 8-(cyclohexyloxy)-1-oxo-2-(2-thienyl)-1*H*-pyrido[2,1-*b*][1,3]benzothiazole-4-carboxylate (37). In a round-bottom flask, 2-thienylacetic anhydride[3] (1.42 g, 5.34 mmol) was added to the benzothiazole acrylate **30**[2] (1.00 g, 2.67 mmol), and the resulting mixture was introduced into a preheated oil bath at 110 °C and stirred for 1h. Subsequently, the reaction mixture was allowed to cool to room temperature and triturated in EtOH. Following filtration, derivative **37** was obtained as a yellow solid in 80% yield. ^{1}H NMR (400 MHz, DMSO-d6): δ 8.75 (s, 1H, Ar-H), 8.30 (s, 1H, Ar-H), 7.90 (d, J = 8.8 Hz, 1H, Ar-H), 7.70 (d, J = 3.4 Hz, 1H, Ar-H), 7.50 (d, J = 5.6 Hz, 1H, Ar-H), 7.25- 7.05 (m, 2H, Ar-H), 4.50-4.25 (m, 3H, OCH and O$CH_2$$CH_3$), 2.00-1.80 (m, 2H, cyclohexyl-CH_2) 1.75-1.55 (m, 2H, cyclohexyl-CH_2) 1.50-1.20 (m, 9H, cyclohexyl-CH_2 x3 and $OCH_2$$CH_3$).

8-(Cyclohexyloxy)-1-oxo-2-(2-thienyl)-1*H*-pyrido[2,1-*b*][1,3]benzothiazole-4-carboxylic acid (40). Following the general procedure B, starting from compound **37** (time: 2h), compound **40** was obtained as a yellow solid in 99% yield (1.18 g). ^{1}H NMR (400 MHz, DMSO-d6): δ 13.30 (bs, 1H, CO_2H), 8.80 (s, 1H, Ar-H) 8.50 (s, 1H, Ar-H), 7.95 (d, J = 9.0 Hz, 1H, Ar-H), 7.75 (d, J = 3.7 Hz, 1H, Ar-H), 7.50 (d, J = 5.1 Hz, 1H, Ar-H), 7.20 (dd, J = 2.7 and 2.0 Hz, 1H, Ar-H), 7.10 (t, J = 4.1 Hz, 1H, Ar-H), 4.50-4.30 (m, 1H, OCH), 2.00-1.80 (m, 2H, cyclohexyl-CH_2), 1.75-1.55 (m, 2H, cyclohexyl-CH_2), 1.50-1.20 (m, 6H, cyclohexyl-CH_2 x3).

Methyl *N*-[[8-(cyclohexyloxy)-1-oxo-2-(2-thienyl)-1*H*-pyrido[2,1-*b*][1,3]benzothiazol-4-yl]carbonyl)-L-tyrosinate (43). Following the general procedure C, starting from compound **40** and using tyrosine methyl ester hydrochloride, TBTU and DIPEA (time: 1.5h), compound **43** was obtained after filtration and purification by flash chromatography column eluting with $CHCl_3$/MeOH

99:1 as a yellow solid in 30% yield (0.41 g). ^{1}H NMR (400 MHz, DMSO-d6): δ 9.20 (s, 1H, OH), 9.00 (d, *J* = 7.1 Hz, 1H, NH), 8.85-8.75 (m, 2H, Ar-H), 7.95-7.80 (m, 2H, Ar-H), 7.50 (d, *J* = 5.2 Hz, 1H, Ar-H), 7.30-7.20 (m, 2H, Ar-H), 7.15-7.00 (m, 2H, Ar-H), 6.70-6.60 (m, 2H, Ar-H). 4.70-4.60 (m, 1H, CH), 4.50-4.40 (m, 1H, OCH), 3.70 (s, 3H, CO_2CH_3), 3.10-2.95 (m, 2H, CH_2), 2.00-1.80 (m, 2H, cyclohexyl-CH_2), 1.75-1.55 (m, 2H, cyclohexyl-CH_2), 1.50-1.20 (m, 6H, cyclohexyl-CH_2 x3).

Methyl *N*-{[8-(cyclohexyloxy)-1-oxo-2-phenyl-1*H*-pyrido[2,1-*b*][1,3]benzothiazol-4-yl]carbonyl}-L-phenylalaninate (44). Following the general procedure C, starting from compound **41**[4] and using phenylalanine methyl ester hydrochloride, TBTU and DIPEA (time: 4h), compound **44** was obtained after filtration and purification by flash chromatography column eluting with $CHCl_3$/MeOH 98:2 as a yellow solid in 60% yield (0.28 g). ^{1}H NMR (400 MHz, DMSO-d6): δ 9.00 (d, *J* = 7.7 Hz, 1H, NH), 8.80 (d, *J* = 2.3 Hz, 1H, Ar-H), 8.40 (s, 1H, Ar-H), 7.85 (d, *J* = 8.8 Hz, 1H, Ar-H), 7.75-7.65 (m, 2H, Ar-H), 7.50-7.30 (m, 3H, Ar-H), 7.25-7.10 (m, 6H, Ar-H), 4.70-4.60 (m, 1H, CH), 4.40-4.25 (m, 1H, OCH), 3.55 (s, 1H, CO_2CH_3), 3.15-3.00 (m, 2H, CH_2), 2.00-1.80 (m, 2H, cyclohexyl-CH_2), 1.75-1.55 (m, 2H, cyclohexyl-CH_2), 1.50-1.20 (m, 6H, cyclohexyl-CH_2 x3).

Methyl *N*-[8-(cyclohexyloxy)-1-oxo-1*H*-pyrido[2,1-*b*][1,3]benzothiazol-4-yl]carbonyl)-L tyrosinate (45). Following the general procedure C, **42**[5] and using tyrosine methyl ester hydrochloride, TBTU and DIPEA (time: 2h), compound **45** was obtained after extraction and purification by flash chromatography column eluting with $CHCl_3$/MeOH 97:3 as a yellow solid in 27% yield (0.19 g). ^{1}H NMR (400 MHz, DMSO-d6): δ 9.20 (s, 1H, OH), 8.75-8.65 (m, 2H, NH and Ar-H), 8.45 (d, *J* = 8.8 Hz, 1H, Ar-H), 7.80 (d, *J* = 8.7 Hz, 1H, Ar-H), 7.15 (dd, *J* = 2.4 and 8.7 Hz, 1H, Ar-H), 7.05-6.95 (m, 2H, Ar-H), 6.60-6.50 (m, 2H, Ar-H), 6.30 (d, *J* = 8.8 Hz, 1H, Ar-H), 4.70-4.60 (m, 1H, CH), 4.50-4.30 (m, 1H, OCH), 3.60 (s, 3H, CO_2CH_3), 3.00-2.80 (m, 2H, CH_2), 2.00-1.80 (m, 2H, cyclohexyl-CH_2), 1.75-1.55 (m, 2H, cyclohexyl-CH_2), 1.50-1.20 (m, 6H, cyclohexyl-CH_2 x3).

***N*-{[8-(cyclohexyloxy)-1-oxo-2-(2-thienyl)-1*H*-pyrido[2,1-*b*][1,3]benzothiazol-4-yI]carbonyI}-L-tyrosine (25)**. Following the general procedure D, starting from **43** (time: 1h), compound **25** was obtained after purification by crystallization with cyclohexane/EtOAc as a yellow solid in 60% yield. ^{1}H NMR (400 MHz, DMSO-d6): δ 12.95 (bs, 1H, CO_2H), 9.20 (s, 1H, OH), 9.00 (d, *J* = 7.1 Hz, 1H, NH), 8.85-8.75 (m, 2H, Ar-H), 7.95-7.80 (m, 2H, Ar-H), 7.50 (d, *J* = 5.2 Hz, 1H, Ar-H), 7.30-7.20 (m, 2H, Ar-H), 7.15-7.00 (m, 2H, Ar-H), 6.70-6.60 (m, 2H, Ar-H), 4.70-4.60 (m, 1H, CH), 4.50-4.40 (m, 1H, OCH), 3.10 (dd, *J* = 4.5 and 14.0 Hz, 1H, CH_2 x ½), 3.00-2.90 (m, 1H, CH_2 x ½), 2.00-1.80 (m, 2H, cyclohexyl-CH_2), 1.75-1.55 (m, 2H, cyclohexyl-CH_2), 1.50-1.20 (m, 6H cyclohexyl-CH_2 x3). ^{13}C NMR (100 MHz, DMSO-d6): δ 173.82, 164.35, 159.95, 156.43, 156.32, 150.62, 138.87, 137.14, 130.47, 129.90, 129.77, 128.41, 126.87, 124.44, 123.22, 121.10, 116.48, 116.38, 115.48, 107.91, 105.58, 75.70, 55.23, 36.18, 31.54, 23.43, 21.21. HPLC, Method A: r_t = 9.64 min. HRMS calculated for $C_{31}H_{28}N_2O_6S_2$ $[M-H]^-$ 587.1316; found 587.1324.

***N*-{[8-(cyclohexyloxy)-1-oxo-2-phenyl-1*H*-pyrido[2,1-*b*][1,3]benzothiazol-4-yl]carbonyl}-L phenylalanine (26).** Following the general procedure D, starting from **44** (time: 3h), compound **26** was obtained after purification by flash column chromatography eluting with $CHCI_3$/MeOH 90:10 as a yellow solid in 30% yield. ^{1}H NMR (400 MHz, DMSO-d6): δ 13.00 (bs, 1H, CO_2H), 9.00-8.80 (m, 2H, Ar-H), 8.50 (s, 1H, Ar-H), 7.90 (d, *J* = 8.3 Hz, 1H, Ar-H), 7.75-7.65 (m, 2H, Ar-H), 7.50-7.30 (m, 3H, Ar-H), 7.25-7.10 (m, 5H, Ar-H), 4.70-4.60 (m, 1H, CH), 4.40-4.30 (m, 1H, OCH), 3.15-3.05 (m, 1H, CH_2 x ½), 3.00-2.95 (m, 1H, CH_2 x ½), 2.00-1.80 (m, 2H, cyclohexyl-CH_2), 1.75-1.55 (m, 2H, cyclohexyl-CH_2), 1.50-1.20 (m, 6H, cyclohexyl-CH_2 x3). ^{13}C NMR (100 MHz, DMSO-d6): δ 173.62, 164.52, 161.31, 156.35, 151.91, 138.97, 138.51, 136.77, 133.88, 129.46, 129.35, 128.68, 128.46, 127.82, 126.85, 123.10, 122.43, 120.90, 116.54, 107.58, 105.34, 75.71, 54.75, 36.86, 31.58, 25.52, 23.47. HPLC, Method A: r_t = 11.25 min. HRMS calculated for $C_{33}H_{30}N_2O_5S$ $[M-H]^-$ 565.1802; found 565.1809.

***N*-{[8-(cyclohexyloxy)-1-oxo-1*H*-pyrido[2,1-*b*][1,3]benzothiazol-4-yl]carbonyl}-L-tyrosine (27).** Following the general procedure D, starting from **45** (time: 1h), compound **27** was obtained after purification by crystallization with cyclohexane/EtOAc as a yellow solid in 50% yield. ^{1}H NMR (400 MHz, DMSO-d6): δ 12.95 (bs, 1H, CO_2H), 9.20 (bs, 1H, OH), 8.75 (s, 1H, Ar-H), 8.65 (d, *J* = 7.8 Hz, 1H, NH), 8.25 (d, *J* = 8.8 Hz, 1H, Ar-H), 7.80 (d, *J* = 8.7 Hz, 1H, Ar-H), 7.20 (d, *J* = 8.7, 1H, Ar-H), 7.10-7.00 (m, 2H, Ar-H), 6.60-6.50 (m, 2H, Ar-H), 6.40 (d, *J* = 8.8 Hz, 1H, Ar-H), 4.60-4.45 (m, 1H, CH), 4.35-4.25 (m, 1H, OCH), 3.05 (dd, *J* = 4.5 and 13.6 Hz, 1H, CH_2 x ½), 3.00-2.90 (m, 1H, CH_2 x ½), 2.00-1.80 (m, 2H, cyclohexyl-CH_2), 1.75-1.55 (m, 2H, cyclohexyl-CH_2), 1.50-1.20 (m, 6H, cyclohexyl-CH_2 x3). ^{13}C NMR (100 MHz, DMSO-d6): δ 173.83, 164.41, 162.21, 156.30, 156.27, 153.17, 138.61, 135.72, 130.42, 128.52, 123.03, 120.51, 116.15, 115.45 111.69, 107.24, 105.28, 75.70, 55.23, 35.91, 31.59, 25.51, 23.54. HPLC, Method A: r_t = 8.09 min. HRMS calculated for $C_{27}H_{26}N_2O_6S$ $[M-H]^-$ 505.1439; found 505.1447.

Ethyl 8-hydroxy-1-oxo-2-phenyl-1*H*-pyrido[2,1-*b*][1,3]benzothiazole-4-carboxylate (46). Under N_2 atmosphere, to a solution of compound **38** (1.00 g, 2.23 mmol) in dry CH_2Cl_2 (50 mL) 1M BBr_3 solution in CH_2Cl_2 (8.93 mL, 8.93 mmol) was dripped at 0 °C and then the reaction was stirred at the same temperature for 1h. Subsequently, MeOH (10 mL) was added at 0 °C to quench the excess of BBr_3, and then the mixture was poured into ice/water and filtered under vacuum. The filtrate was extracted with EtOAc (x3) and the organic phase was washed with brine, dried over Na_2SO_4, and evaporated to dryness to afford derivative **46** as a yellow solid in 70% yield (0.57 g). ^{1}H NMR (400 MHz, DMSO-*d6*): *δ* = 10.21 (s, 1H, OH), 8.76 (d, *J* = 1.5 Hz, 1H, Ar-H), 8.05 (s, 1H, Ar-H), 7.91 (d, *J* = 8.5 Hz, 1H, Ar-H), 7.73 (d, *J* = 7.4 Hz, 2H, Ar-H), 7.47 (t, *J* = 7.5 Hz, 2H, Ar-H), 7.31 (t, *J* = 7.2 Hz, 1H, Ar-H), 7.08 (d, *J* = 7.2 Hz, 1H, Ar-H), 4.31 (q, *J* = 7.0 Hz, 2H, O$CH_2$$CH_3$), 1.29 (t, *J* = 7.0 Hz, 3H, O$CH_2$$CH_3$).

Ethyl 8-[2-(diethylamino)ethoxy]-1-oxo-2-phenyl-1*H*-pyrido[2,1-*b*][1,3]benzothiazole-4-carboxylate (47). Following the general procedure A, starting from compound **46** and using 2-(diethylamino)ethyl chloride hydrochloride as electrophile (temperature: 80 °C; time: 2h), compound **47** was obtained as a yellow solid in 75% yield (0.17 g). ^{1}H NMR (400 MHz, $CDCl_3$): *δ* = 9.06 (d, *J* = 2.4 Hz, 1H, Ar-H), 8.20 (s, 1H, Ar-H), 7.73-7.68 (m, 2H, Ar-H), 7.61 (d, *J* = 7.7 Hz, 1H, Ar-H), 7.46 (t, *J* = 7.7 Hz, 2H, Ar-H), 7.35 (t, *J* = 7.4 Hz, 1H, Ar-H), 7.12 (dd, *J* = 2.4 and 8.9 Hz, 1H, Ar-H), 4.46 (q, *J* = 6.9 Hz, 2H, O$CH_2$$CH_3$), 4.12 (t, *J* = 5.9 Hz, 2H, O$CH_2$$CH_2$N), 2.90 (t, *J* = 5.8 Hz, 2H, O$CH_2$$CH_2$N), 2.63 (q, *J* = 7.1 Hz, 4H, N$CH_2$$CH_3$ x2), 1.40 (t, *J* = 6.8 Hz, 3H, O$CH_2$$CH_3$), 1.03 (t, *J* = 7.1 Hz, 6H, N$CH_2$$CH_3$ x2).

8-[2-(Diethylamino)ethoxy]-1-oxo-2-phenyl-1*H*-pyrido[2,1-*b*][1,3]benzothiazole-4-carboxylic acid (48). Following the general procedure B, starting from compound **47** (time: 2h), compound **48** was obtained as a yellow solid in 68% yield (0.29 g). ^{1}H NMR (400 MHz, DMSO-*d6*): *δ* = 13.55 (bs, 1H, CO_2H), 8.80-8.77 (m, 1H, Ar-H), 8.04 (s, 1H, Ar-H), 7.76 (d, *J* = 7.3 Hz, 2H, Ar-H), 7.63-7.60 (m, 1H, Ar-H), 7.46 (t, *J* = 7.5 Hz, 2H, Ar-H), 7.33 (t, *J* = 7.4 Hz, 1H, Ar-H), 7.04-7.00 (m, 1H, Ar-H), 4.29-4.25 (m, 2H, O$CH_2$$CH_2$N), 3.24-3.19 (m, 2H, O$CH_2$$CH_2$N), 3.00-2.93 (m, 4H, N$CH_2$$CH_3$ x2), 1.24-1.17 (m, 6H, N$CH_2$$CH_3$ x2).

4-(Aminosulfonyl)phenyl 8-[2-(diethylamino)ethoxy]-1-oxo-2-phenyl-1*H*-pyrido[2,1-*b*][1,3]benzothiazole-4-carboxylate (28). Following the general procedure C, starting from compound **48** and using 4-hydroxybenzenesulfonamide, BOP and Et_3N (time: 3h), compound **28** was obtained after filtration and purification by flash chromatography column eluting with $CHCl_3$/MeOH 95:5 as a yellow solid in 20% yield (0.10 g). ^{1}H NMR (400 MHz, DMSO-*d6*): *δ* = 8.80 (d, *J* = 2.1 Hz, 1H, Ar-H), 8.31 (s, 1H, Ar-H), 8.02 (d, *J* = 8.8 Hz, 1H, Ar-H), 7.95 (d, *J* = 8.7 Hz, 2H, Ar-H), 7.81-7.77 (m, 2H, Ar-H), 7.55 (d, *J* = 8.7 Hz, 2H, Ar-H), 7.51-7.45 (m, 4H, Ar-H and SO_2NH_2), 7.43-7.38 (m, 1H, Ar-H), 7.23 (dd, *J* = 2.1 and 8.8 Hz, 1H, Ar-H), 4.13-4.08 (m, 2H, O$CH_2$$CH_2$N), 2.94-2.85 (m, 2H, O$CH_2$$CH_2$N), 2.67-2.57 (m, 4H, N$CH_2$$CH_3$ x2), 1.01 (t, *J* = 7.0 Hz, 6H, N$CH_2$$CH_3$ x2). ^{13}C

NMR (100 MHz, DMSO-d_6): δ = 162.81, 161.21, 157.91, 156.21, 153.01, 142.17, 139.06, 136.08, 135.56, 129.10, 128.53, 128.06, 127.76, 123.56, 123.08, 122.94, 119.43, 115.67, 105.61, 101.28, 67.03, 51.44, 47.42, 11.96. HPLC, Method A: r_t = 5.017 min. HRMS calculated for $C_{30}H_{29}N_3O_6S_2$ $[M+H]^+$ 592.1571, found 592.15745.

Ethyl 2-bromo-8-(cyclohexyloxy)-1-oxo-1*H*-pyrido[2,1-*b*][1,3]benzothiazole-4-carboxylate (49). To a suspension of **39** (0.48 g, 1.28 mmol) in AcOH (6 mL per mmol), Br_2 (0.08 mL, 1.54 mmol) was added dropwise and then the reaction was stirred at rt for 30 min. Subsequently, 10% Na_2SO_3 solution in H_2O (50 mL) was added to quench the excess of Br_2 and the precipitate was filtered under vacuum to afford compound **49** as an orange solid in 85% yield. ^{1}H NMR (400 MHz, $CDCl_3$): δ = 9.01 (d, J = 4.2 Hz, 1H, Ar-H), 8.47 (s, 1H, Ar-H), 7.64 (d, J = 8.7 Hz, 1H, Ar-H), 7.18 (dd, J = 2.4 and 8.7 Hz, 1H, Ar-H), 4.49-4.40 (m, 3H, O$CH_2$$CH_3$ and OCH), 2.07-2.01 (m, 2H, cyclohexyl-CH_2), 1.82-1.76 (m, 2H, cyclohexyl-CH_2), 1.60-1.52 (m, 3H, cyclohexyl-CH_2 and cyclohexyl-CH_2 x$^1/_2$), 1.49-1.31 (m, 6H, OCH_2*CH_3*, cyclohexyl-CH_2 and cyclohexyl-CH_2 x$^1/_2$).

Ethyl 8-(cyclohexyloxy)-2-[(2-morpholin-4-ylethyl)amino]-1-oxo-1*H*-pyrido[2,1-*b*][1,3]benzothiazole-4-carboxylate (50). Following the creation of a vacuum within a three-necked flask, under a N_2 atmosphere, dry toluene (15 mL) was added to a mixture comprising compound **49** (0.47 mmol, 0.21 g.), 4-(2-aminoethyl)morpholine (1.40 mmol, 0.17 mL), BINAP (0.014 mmol, 0.008 g), $Pd(OAc)_2$ (0.014 mmol, 0.003 g), and NaO*t*Bu (0.94 mmol, 0.09 g) and the reaction was stirred at reflux for 12h. Subsequently, the mixture was filtered over Celite, and the filtrate was evaporated to dryness to give an oil that was purified by flash chromatography column eluting with $CHCl_3$/MeOH 98:2 to give compound **50** as a yellow solid in 55% yield. ^{1}H NMR (400 MHz, $CDCl_3$): δ = 8.99 (d, J = 2.3 Hz, 1H, Ar-H), 7.55 (d, J = 8.6 Hz, 1H, Ar-H), 7.11 (dd, J = 2.4 and 8.6 Hz, 1H, Ar-H), 7.03 (s, 1H, Ar-H), 5.27 (bs, 1H, NH), 4.49-4.43 (m, 3H, O$CH_2$$CH_3$ and OCH), 3.81-3.74 (m, 4H, morpholine-CH_2 x2), 3.27 (q, J = 5.7 Hz, 2H, NH$CH_2$$CH_2$N), 2.76 (t, J = 5.8 Hz, 2H, NH$CH_2$$CH_2$N), 2.57-2.51 (m, 4H, morpholine-CH_2 x2), 2.07-2.01 (m, 2H, cyclohexyl-CH_2), 1.85-1.77 (m, 2H, cyclohexyl-CH_2), 1.65-1.58 (m, 4H, cyclohexyl-CH_2 x2), 1.52-1.45 (m, 5H, cyclohexyl-CH_2 and OCH_2*CH_3*).

8-(Cyclohexyloxy)-2-[(2-morpholin-4-ylethyl)amino]-1-oxo-1*H*-pyrido[2,1-*b*][1,3]benzothiazole-4-carboxylic acid (51). Following the general procedure B, starting from compound **50** (time: 2h), compound **51** was obtained as a yellow solid in 92% yield (0.13 g). ^{1}H NMR (400 MHz, DMSO-d_6): δ = 13.55 (s, 1H, CO_2H), 8.80-8.78 (m, 1H, Ar-H), 7.77 (d, J = 8.4 Hz, 1H, Ar-H), 7.14 (d, J = 7.2 Hz, 1H, Ar-H), 6.98 (s, 1H, Ar-H), 5.45 (bs, 1H, NH), 4.45-4.41 (m, 1H, OCH), 3.59-3.53 (m, 4H, morpholine-CH_2 x2), 3.26-3.21 (m, 2H, NH$CH_2$$CH_2$N), 2.61-2.56 (m, 2H, NH$CH_2$$CH_2$N), 2.49-2.39 (m, 4H, morpholine-CH_2 x2), 2.02-1.89 (m, 2H, cyclohexyl-CH_2), 1.79-1.73 (m, 2H, cyclohexyl-CH_2), 1.67-1.23 (m, 6H, cyclohexyl-CH_2 x3).

4-(Aminosulfonyl)phenyl 8-(cyclohexyloxy)-2-[(2-morpholin-4-ylethyl)amino]-1-oxo-1*H*-pyrido[2,1-*b*][1,3]benzothiazole-4-carboxylate (29). Following the general procedure C, starting from compound **51** and using 4-hydroxybenzenesulfonamide, BOP and Et_3N (time: 1h), compound **29** was obtained after filtration and purification by flash chromatography column eluting with $CHCl_3$/MeOH 93:7 as a yellow solid in 20% yield (0.06 g). ^{1}H NMR (400 MHz, DMSO-d_6): δ = 8.81 (d, J = 1.8 Hz, 1H, Ar-H), 7.96-7.87 (m, 3H, Ar-H), 7.54 (d, J = 8.6 Hz, 2H, Ar-H), 7.49 (bs, 2H, SO_2NH_2), 7.21 (dd, J = 2.1 and 8.7 Hz, 1H, Ar-H), 7.11 (s, 1H, Ar-H), 5.61 (bs, 1H, NH), 4.46-4.38 (m, 1H, OCH), 3.65-3.59 (m, 4H, morpholine-CH_2 x2), 3.30-3.24 (m, 2H, NH$CH_2$$CH_2$N), 2.69-2.61 (m, 2H, NH$CH_2$$CH_2$N), 2.49-2.38 (m, 4H, morpholine-CH_2 x2), 2.03-1.95 (m, 2H, cyclohexyl-CH_2), 1.79-1.71 (m, 2H, cyclohexyl-CH_2), 1.59-1.30 (m, 6H, cyclohexyl-CH_2 x3). ^{13}C NMR (100 MHz, DMSO-d_6): δ = 163.62, 158.38, 156.57, 153.31, 142.29, 141.36, 138.65, 134.16, 127.95, 123.56, 123.25, 119.51, 116.49, 107.30, 105.20, 101.62, 75.87, 66.89, 56.87, 53.67 (2C), 31.70, 25.63, 23.68. HPLC, Method A: r_t = 5.767 min. HRMS calculated for $C_{30}H_{34}N_4O_7S_2$ $[M+H]^+$ 627.1942, found 627.19547.

*Determination of λ_{max} of fluorescence emission of compound **1** (autofluorescence)*

Firstly, the adsorption UV-Vis spectrum (280-600 nm) of compound **1** (1 µM solution in H_2O with 0.01% DMSO) was determined using a microplate reader (Infinite M200 Pro TECAN) on a Corning® 96-well UV-transparent microplate. The excitation wavelength with the greatest intensity for compound **1** was 392 nm, which is characteristic of the PBTZ core. Subsequently, using the λ_{max} of excitation (392 nm), the fluorescence excitation spectrum was recorded (400-800 nm) using a microplate reader (Infinite M200 Pro TECAN) on a Costar 96 Flat Bottom Black Polystyrene microplate. The experimental setup was configured with the following parameters: emission wavelength step size: 2 nm; gain 100; number of flashes: 25; Z-position: 20,000 µm. The emission λ_{max} for compound **1** was observed to be 438 nm.

Supplementary Table

Table S1. Chemical structures of the tested compounds.

Code	Chemical structures	SMILES
**1** (Felicetti et al., 2021)	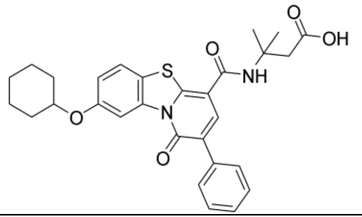	O=C1N(C(S2)=C(C(NC(C)(C)CC(O)=O)=O)C=C1C3=CC=CC=C3)C4=C2C=CC(OC5CCCCC5)=C4
**2** (Cannalire et al., 2019)	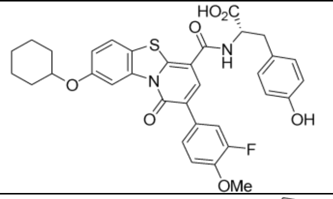	O=C1N(C(S2)=C(C(N[C@H](C(O)=O)CC3=CC=C(O)C=C3)=O)C=C1C4=CC=C(OC)C(F)=C4)C5=C2C=CC(OC6CCCCC6)=C5
**3** (Cannalire et al., 2020)	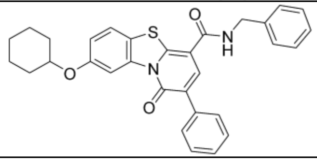	O=C1N(C(S2)=C(C(NCC3=CC=CC=C3)=O)C=C1C4=CC=CC=C4)C5=C2C=CC(OC6CCCCC6)=C5
**4** (Felicetti et al., 2021)	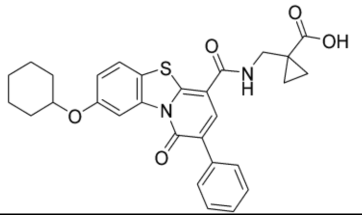	O=C1N(C(S2)=C(C(NCC3(CC3)C(O)=O)=O)C=C1C4=CC=CC=C4)C5=C2C=CC(OC6CCCCC6)=C5
**5** (Cannalire et al., 2019)	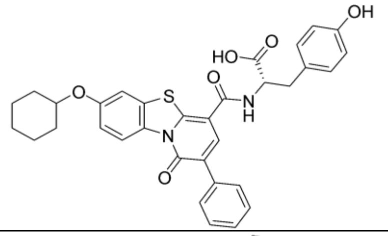	O=C1N(C(S2)=C(C(N[C@H](C(O)=O)CC3=CC=C(O)C=C3)=O)C=C1C4=CC=CC=C4)C5=C2C=C(OC6CCCCC6)C=C5
**6** (Felicetti et al., 2021)	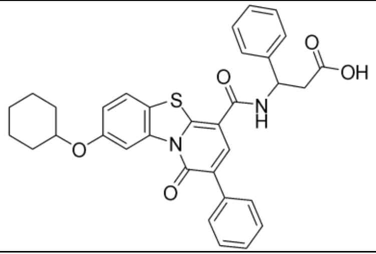	O=C1N(C(S2)=C(C(NC(C3=CC=CC=C3)CC(O)=O)=O)C=C1C4=CC=CC=C4)C5=C2C=CC(OC6CCCCC6)=C5

**7**<br>(Felicetti et al., 2021)	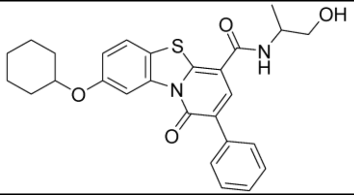	O=C1N(C(S2)=C(C(NC(C)CO)=O)C=C1C3=CC=CC=C3)C4=C2C=CC(OC5CCCCC5)=C4
**8**<br>(Felicetti et al., 2021)	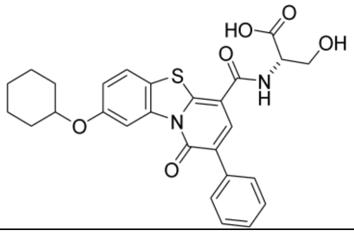	O=C1N(C(S2)=C(C(N[C@H](C(O)=O)CO)=O)C=C1C3=CC=CC=C3)C4=C2C=CC(OC5CCCCC5)=C4
**9**<br>(Cannalire et al., 2019)	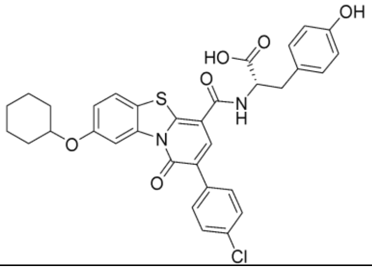	O=C1N2C3=CC(OC4CCCCC4)=CC=C3SC2=C(C(N[C@H](C(O)=O)CC5=CC=C(O)C=C5)=O)C=C1C6=CC=C(Cl)C=C6
**10**<br>(Cannalire et al., 2020)	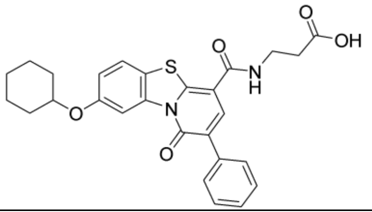	O=C1N(C(S2)=C(C(NCCC(O)=O)=O)C=C1C3=CC=CC=C3)C4=C2C=CC(OC5CCCCC5)=C4
**11**<br>(Felicetti et al., 2021)	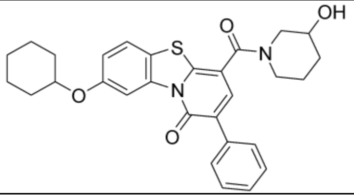	O=C1N(C(S2)=C(C(N3CCCC(O)C3)=O)C=C1C4=CC=CC=C4)C5=C2C=CC(OC6CCCCC6)=C5
**12**<br>(Felicetti et al., 2021)	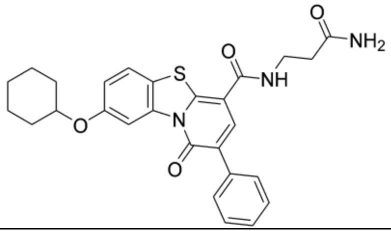	O=C1N(C(S2)=C(C(NCCC(N)=O)=O)C=C1C3=CC=CC=C3)C4=C2C=CC(OC5CCCCC5)=C4
**13**<br>(Cannalire et al., 2020)	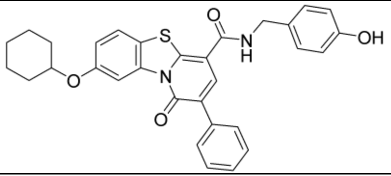	O=C1N(C(S2)=C(C(NCC3=CC=C(O)C=C3)=O)C=C1C4=CC=CC=C4)C5=C2C=CC(OC6CCCCC6)=C5
**14**<br>(Cannalire et al., 2019)	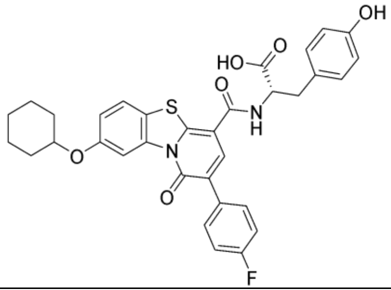	O=C1N(C(S2)=C(C(N[C@H](C(O)=O)CC3=CC=C(O)C=C3)=O)C=C1C4=CC=C(F)C=C4)C5=C2C=CC(OC6CCCCC6)=C5

**15**<br>(Cannalire et al., 2019)	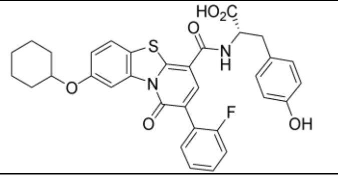	O=C1N(C(S2)=C(C(N[C@H](C(O)=O)CC3=CC=C(O)C=C3)=O)C=C1C4=CC=CC=C4F)C5=C2C=CC(OC6CCCCC6)=C5
**16**<br>(Cannalire et al., 2019)	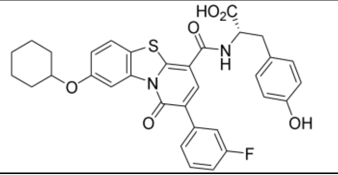	O=C1N(C(S2)=C(C(N[C@H](C(O)=O)CC3=CC=C(O)C=C3)=O)C=C1C4=CC=CC(F)=C4)C5=C2C=CC(OC6CCCCC6)=C5
**17**<br>(Cannalire et al., 2019)	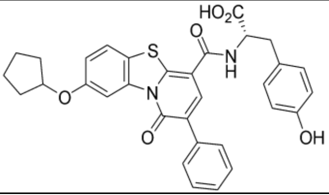	O=C1N(C(S2)=C(C(N[C@H](C(O)=O)CC3=CC=C(O)C=C3)=O)C=C1C4=CC=CC=C4)C5=C2C=CC(OC6CCCC6)=C5
**18**<br>(Cannalire et al., 2019)	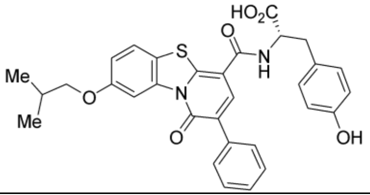	O=C1N(C(S2)=C(C(N[C@H](C(O)=O)CC3=CC=C(O)C=C3)=O)C=C1C4=CC=CC=C4)C5=C2C=CC(OCC(C)C)=C5
**19**<br>(Felicetti et al., 2021)	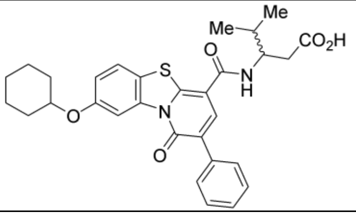	O=C1N(C(S2)=C(C(NC(C(C)C)CC(O)=O)=O)C=C1C3=CC=CC=C3)C4=C2C=CC(OC5CCCCC5)=C4
**20**<br>(Felicetti et al., 2021)	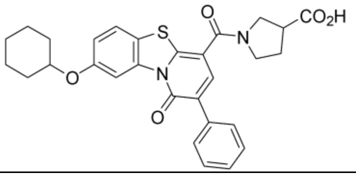	O=C1N(C(S2)=C(C(N3CCC(C(O)=O)C3)=O)C=C1C4=CC=CC=C4)C5=C2C=CC(OC6CCCCC6)=C5
**21**<br>(Felicetti et al., 2021)	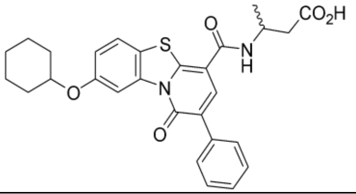	O=C1N(C(S2)=C(C(NC(C)CC(O)=O)=O)C=C1C3=CC=CC=C3)C4=C2C=CC(OC5CCCCC5)=C4
**22**	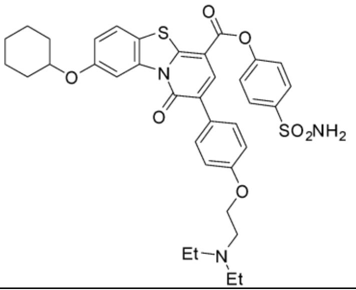	O=C1N(C(S2)=C(C(OC3=CC=C(S(=O)(N)=O)C=C3)=O)C=C1C4=CC=C(OCCN(CC)CC)C=C4)C5=C2C=CC(OC6CCCCC6)=C5
**23**	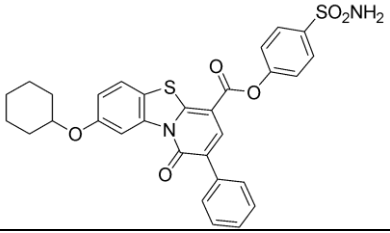	O=C1N(C(S2)=C(C(OC3=CC=C(S(=O)(N)=O)C=C3)=O)C=C1C4=CC=CC=C4)C5=C2C=CC(OC6CCCCC6)=C5

**24**	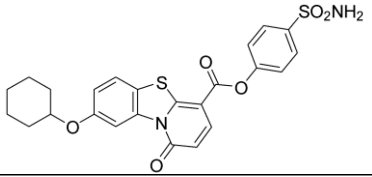	O=C1N(C(S2)=C(C(OC3=CC=C(S(=O)(N)=O)C=C3)=O)C=C1)C4=C2C=CC(OC5CCCCC5)=C4
**25**	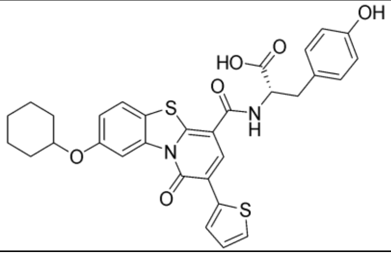	O=C1N2C3=CC(OC4CCCCC4)=CC=C3SC2=C(C(N[C@H](C(O)=O)CC5=CC=C(O)C=C5)=O)C=C1C6=CC=CS6
**26**	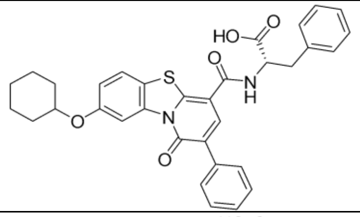	O=C1N(C(S2)=C(C(N[C@H](C(O)=O)CC3=CC=CC=C3)=O)C=C1C4=CC=CC=C4)C5=C2C=CC(OC6CCCCC6)=C5
**27**	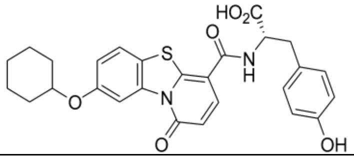	O=C1N(C(S2)=C(C(N[C@H](C(O)=O)CC3=CC=C(O)C=C3)=O)C=C1)C4=C2C=CC(OC5CCCCC5)=C4
**28**	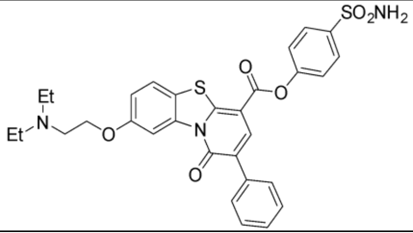	O=C1N2C3=CC(OCCN(CC)CC)=CC=C3SC2=C(C(OC4=CC=C(S(=O)(N)=O)C=C4)=O)C=C1C5=CC=CC=C5
**29**	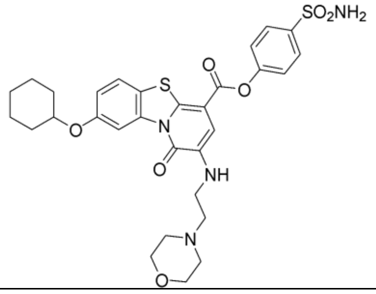	O=C1N(C(S2)=C(C(OC3=CC=C(S(=O)(N)=O)C=C3)=O)C=C1NCCN4CCOCC4)C5=C2C=CC(OC6CCCCC6)=C5

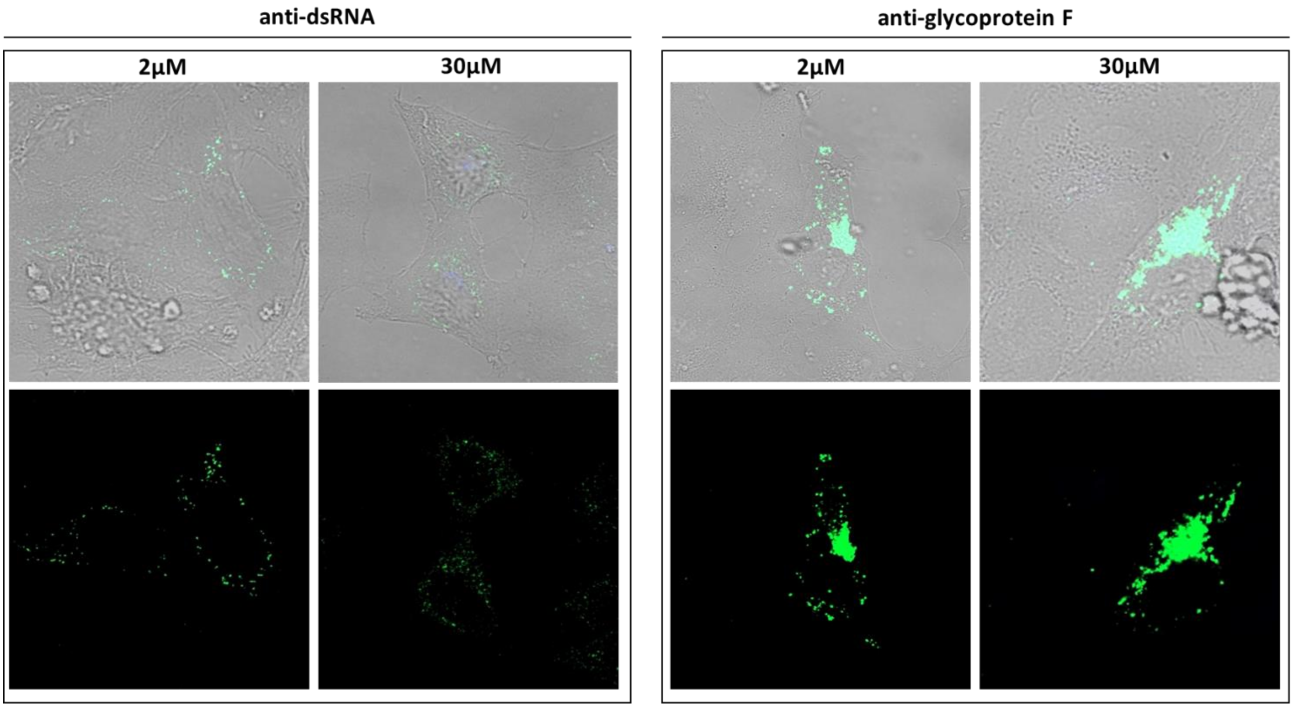

Figure S1. Compound 1 at different doses inhibited viral replication at different phases for RSV-A2. Compound 1 did not inhibit nucleic acid replication and viral protein production of RSV-A2 after 24h post-infection. Cells were treated with EC_{50} (2μM) or 2-fold EC_{90} dose (30μM) of compound 1 and concurrently infected with RSV-A2. The dsRNA (left panel) and viral glycoprotein F (right panel) are visualized in green, compound 1 in blue, via with confocal laser microscopy. The pictures are representative of ≥20 images acquired per condition. Magnification, 630X.

References

1. Cannalire, R.; Tarantino, D.; Piorkowski, G.; Carletti, T.; Massari, S.; Felicetti, T.; Barreca, M.L.; Sabatini, S.; Tabarrini, O.; Marcello, A.; et al. Broad Spectrum Anti-Flavivirus Pyridobenzothiazolones Leading to Less Infective Virions. *Antiviral Research* **2019**, *167*, 6–12, doi:10.1016/j.antiviral.2019.03.004.
2. Felicetti, T.; Burali, M.S.; Gwee, C.P.; Ki Chan, K.W.; Alonso, S.; Massari, S.; Sabatini, S.; Tabarrini, O.; Barreca, M.L.; Cecchetti, V.; et al. Sustainable, Three-Component, One-Pot Procedure to Obtain Active Anti-Flavivirus Agents. *European Journal of Medicinal Chemistry* **2021**, *210*, 112992, doi:10.1016/j.ejmech.2020.112992.
3. Smith, S.R.; Fallan, C.; Taylor, J.E.; McLennan, R.; Daniels, D.S.B.; Morrill, L.C.; Slawin, A.M.Z.; Smith, A.D. Asymmetric Isothiourea-Catalysed Formal [3+2] Cycloadditions of Ammonium Enolates with Oxaziridines. *Chemistry* **2015**, *21*, 10530–10536, doi:10.1002/chem.201501271.
4. Tarantino, D.; Cannalire, R.; Mastrangelo, E.; Croci, R.; Querat, G.; Barreca, M.L.; Bolognesi, M.; Manfroni, G.; Cecchetti, V.; Milani, M. Targeting Flavivirus RNA Dependent RNA Polymerase through a Pyridobenzothiazole Inhibitor. *Antiviral Research* **2016**, *134*, 226–235, doi:10.1016/j.antiviral.2016.09.007.
5. Manfroni, G.; Meschini, F.; Barreca, M.L.; Leyssen, P.; Samuele, A.; Iraci, N.; Sabatini, S.; Massari, S.; Maga, G.; Neyts, J.; et al. Pyridobenzothiazole Derivatives as New Chemotype Targeting the HCV NS5B Polymerase. *Bioorg Med Chem* **2012**, *20*, 866–876, doi:10.1016/j.bmc.2011.11.061.
